# Recruitment and Expansion of Tregs Cells in the Tumor Environment—How to Target Them?

**DOI:** 10.3390/cancers13081850

**Published:** 2021-04-13

**Authors:** Justine Cinier, Margaux Hubert, Laurie Besson, Anthony Di Roio, Céline Rodriguez, Vincent Lombardi, Christophe Caux, Christine Ménétrier-Caux

**Affiliations:** 1 University of Lyon, Claude Bernard Lyon 1 University, INSERM U-1052, CNRS 5286 Centre Léon Bérard, Cancer Research Center of Lyon (CRCL), 69008 Lyon, France; justine.cinier@lyon.unicancer.fr (J.C.); margaux.hubert@lyon.unicancer.fr (M.H.); laurie.besson@lyon.unicancer.fr (L.B.); anthony.diroio@lyon.unicancer.fr (A.D.R.); celine.rodriguez@lyon.unicancer.fr (C.R.); christophe.caux@lyon.unicancer.fr (C.C.); 2 Institut de Recherche Servier, 125 Chemin de Ronde, 78290 Croissy-sur-Seine, France; vincent.lombardi@servier.com

**Keywords:** regulatory T cells, tumor microenvironment, recruitment, proliferation, stability, therapeutic targeting

## Abstract

**Simple Summary:**

The immune response against cancer is generated by effector T cells, among them cytotoxic CD8^+^ T cells that destroy cancer cells and helper CD4^+^ T cells that mediate and support the immune response. This antitumor function of T cells is tightly regulated by a particular subset of CD4^+^ T cells, named regulatory T cells (Tregs), through different mechanisms. Even if the complete inhibition of Tregs would be extremely harmful due to their tolerogenic role in impeding autoimmune diseases in the periphery, the targeted blockade of their accumulation at tumor sites or their targeted depletion represent a major therapeutic challenge. This review focuses on the mechanisms favoring Treg recruitment, expansion and stabilization in the tumor microenvironment and the therapeutic strategies developed to block these mechanisms.

**Abstract:**

Regulatory T cells (Tregs) are present in a large majority of solid tumors and are mainly associated with a poor prognosis, as their major function is to inhibit the antitumor immune response contributing to immunosuppression. In this review, we will investigate the mechanisms involved in the recruitment, amplification and stability of Tregs in the tumor microenvironment (TME). We will also review the strategies currently developed to inhibit Tregs’ deleterious impact in the TME by either inhibiting their recruitment, blocking their expansion, favoring their plastic transformation into other CD4^+^ T-cell subsets, blocking their suppressive function or depleting them specifically in the TME to avoid severe deleterious effects associated with Treg neutralization/depletion in the periphery and normal tissues.

## 1. Introduction

Regulatory T cells (Tregs) are a subset of immunosuppressive CD4^+^ T lymphocytes characterized by the expression of the lineage-specifying transcription factor forkhead box P3 (FOXP3). Tregs are actively engaged in the maintenance of immunological self-tolerance by suppressing self-reacting T cells, preventing autoimmunity and limiting chronic inflammatory situations. Indeed, in humans, the loss-of-function mutation in the *FOXP3* gene impairs Treg development and causes a breach in self-tolerance, leading to a severe autoimmune syndrome named immune deficiency poly-endocrinopathy enteropathy X-linked (IPEX) syndrome [1].

### 1.1. Natural and Peripherally—Induced Tregs

Two types of Tregs, the natural Tregs (nTregs) and induced Tregs (iTregs), have been defined based on their site of differentiation. nTregs, also named thymic Tregs (tTregs) develop in the thymus via stimulation by self-antigens (Ag) presented by thymic epithelial cells. They undergo maturation in the thymus and are exported to peripheral tissues to play their role in immunological tolerance with a highly diverse T cell receptor (TCR) repertoire [2]. nTregs represent the major Treg populations of secondary lymphoid organs. iTregs, also named peripherally induced Tregs (pTregs), differentiate in the periphery from naive or conventional CD4^+^ T cells in the presence of sub-optimally activated dendritic cells (DC), sub-immunogenic doses of agonist peptide, the mucosal administration of peptide and/or in the presence of appropriate cytokines such as Tumor growth factor (TGF)-β and interleukin (IL)-2 [3,4,5,6,7]. Apart from these two major populations, T-regulatory type 1 (Tr1) cells are generated in the periphery from naive CD4^+^ T cells independently of FOXP3 and secrete high amounts of IL-10 that also contribute to immunosuppression (for review, [8]) While the generation of nTregs is required to prevent autoimmune responses against self-Ag, iTregs are generated in response to foreign Ag, such as intestinal flora, food allergens or pathogens, that trigger inflammation [9]. 

In this context, both Treg subsets can be observed in the tumor as tumor dying cells could release, in the tumor microenvironment (TME), self-Ag, recognized by nTregs, but, also, tumor-specific Ag encoded by mutated genes, also known as neo-Ag, that represent foreign Ag able to be recognized by iTregs. 

Both nTregs and iTregs have similar phenotypic characteristics (as FOXP3 expression) and immunosuppressive functions. Nevertheless, they differ in their stability and in the epigenetic modifications of their DNA, as well as in the expression of certain transcripts or proteins. For instance, the methylation level of the *FOXP3* Treg-specific demethylated region (TSDR) distinguishes iTregs from nTregs, as this region is highly methylated in iTregs and totally demethylated in nTregs and contributes to their stability [10]. In addition, nTregs exhibit a strong expression of Helios [11] compared to iTregs. Interestingly, in the TME, the majority of Tregs express Helios, potentially indicating that tumor-associated (TA) Tregs would be mostly composed of nTregs [12]. Furthermore, recent data in different types of tumors suggest that self-Ag released by dying cancer cells in the TME are recognized by Tregs, inducing their activation into effector Tregs expressing higher levels of activation markers (CTLA-4, TIGIT, TIM-3, ICOS, OX40, 4-1BB and CD39) and presenting a highly proliferative state, compared with Tregs in the peripheral blood or healthy tissues [13,14].

### 1.2. Mechanisms Developed by Tregs to Suppress T Cells

Tregs restrain the activities of effector T cells through different mechanisms, including cell contact and soluble factor secretion (for review [15]) (for more details, see the review from Tay C et al. in the same issue). Briefly, a high expression of interleukin (IL)-2 receptor alpha chain (CD25) by Tregs can deprive the milieu in IL-2, reducing the expansion of conventional CD4^+^ T cells and memory CD8^+^ T cells and their differentiation into effectors. Tregs are also able to secrete immunosuppressive cytokines such as TGF-β, IL-10 and IL-35, known to inhibit the activation of effector CD4^+^ and CD8^+^ T cells. They have also been described to express cytotoxic molecules (granzyme B and perforin), allowing them to directly kill CD8^+^ T cells and natural killer (NK) cells. The constitutive expression of CTLA-4 by Tregs deprives CD4^+^ and CD8^+^ T cells of CD28 costimulatory signals by downregulating CD80 and CD86 expression in Ag-presenting cells (APC) such as DC and B cells. Finally, the regulation of the extracellular nucleotide metabolism by the two transmembrane ecto-nucleotidases CD39 and CD73 also contributes to Tregs’ suppressive function. Murine Tregs co-express both enzymes [16], favoring the metabolism of extracellular ATP released during cell death into AMP by CD39 and into immunosuppressive adenosine by CD73. The engagement of A2A and A2B receptors by adenosine will inhibit CD4^+^ and CD8^+^ T-cell effector functions [17,18]. However, our group recently demonstrated that human Tregs only express CD39, requiring cooperation from CD73^+^ neighbors to generate adenosine, including polyfunctional CD73^+^ effector CD4^+^ T cells representing a privileged target of CD39^+^ Treg suppression [19].

### 1.3. Tregs Infiltrate Tumors and Participate to Cancer Progression

Tregs are found at high frequencies in the tumor tissue of various types of solid tumors, such as breast [20,21] and ovarian [22] carcinoma, renal cell carcinoma (RCC) [23,24], cervix [25] and prostate [26] carcinoma, muscle invasive bladder carcinoma (MIBC) [27], non-small cell lung carcinoma (NSCLC) [28], hepatocellular carcinoma (HCC) [29], pancreatic adenocarcinoma (PDAC) [30], brain tumors [31], head and neck squamous cell carcinoma (HNSCC) [32] and melanoma [33,34]. Their high frequency among CD4^+^ T cells within tumor-infiltrating lymphocytes (TILs) or a high ratio of FOXP3^+^ Tregs to CD8^+^ T cells is associated with a poor prognosis in the majority of solid tumors (for review, [15,35]. However, there are some exceptions, such as colorectal carcinoma (CRC), HNSCC and Hodgkin’s lymphoma (HL), in which a high number of Tregs correlates to a better prognosis [32,36,37,38]. This may be related to the cancer type or Treg localization, as well as to their functional phenotype and plasticity in different cancer environments. For example, in ulcerative colitis and associated CRC, FOXP3^+^ Tregs exhibit suppressive functions, but their high production of effector cytokines, including TNF-α, IFN-γ and IL-17 [39], may also promote T-cell immunity [40]. However, this may also possibly be related to the low FOXP3 expression in activated T cells in some tumors. Indeed, recent data clarified the impact of Tregs in CRC, in which FOXP3^high^ Tregs remain associated with a poor prognosis, whereas FOXP3^low^ T cells, which could be activated effector T cells, are associated with a good prognosis [41]. Either way, except in CRC, HNSCC and HL, it is well-accepted that tumor-associated (TA)-Tregs suppress the tumor immunity and dampen the therapeutic efficacy of immune intervention, as well as conventional cancer therapy, including chemotherapy and radiotherapy. 

### 1.4. Origin of Tregs Present in the TME

The analysis of Tregs in different contexts using a microarray or profiling of the gene expression by RNA sequencing (RNA-seq) highlighted the existence of an important heterogeneity within the Tregs entity. This heterogeneity is based either on their level of activation, their acquisition of memory markers, homing receptors or chemokine receptors but, also, their expression of dedicated transcription factors, all together favoring their capacity to inhibit specifically the different T-cell subsets. 

Through the initial analyses focused on Tregs from blood or lymphoid tissues, the analysis of several non-lymphoid tissues in mice and humans (skin, lung, liver, intestinal mucosa, placenta, brain, adipose tissue, injured muscle, etc.) highlighted the existence of tissue-specific populations that highly differ from lymphoid ones in phenotype but, also, in function (for review [42]). 

TA-Tregs could originate from tissue Treg expansion, from peripheral Treg recruitment or from local differentiation from naive T cells or the conversion of conventional T cells. Several RNA-seq or single-cell RNA sequencing (scRNA-seq) analyses compared Tregs from tumor tissues (breast, NSCLC, CRC and HCC) with those of normal adjacent tissue (NAT) and peripheral blood [43,44,45,46,47]. In breast tumors, peripheral blood Tregs significantly differ from tissue-resident and TA-Tregs, but the two latter are also clearly distinct when compared together [43,48]. This indicates that tissue- and TA-Tregs share similarities resulting from tissue environment imprinting but that TA-Tregs acquire specific patterns shaped by the TME. In addition, the comparison of the TCR repertoire (by TCR-seq) of Tregs from breast and bladder tissues to their tumor counterpart revealed that TA-Tregs possess a private repertoire with no detected clonotype sharing with other CD4^+^ T cells [27,46] suggesting that they do not emerge from the conversion in the TME of effector CD4^+^ T cells. In contrast, Tregs with shared TCRs are found in tumor-draining lymph nodes and tumors [49]. Altogether, these results suggest that TA-Tregs are recruited from the periphery or tumor-draining lymph nodes and might also expand in the TME. 

In this review, we aim to recapitulate the major data from the literature dedicated to the recruitment and expansion of Tregs in the TME, focusing, when possible, on human data. We will also discuss the different strategies targeting Tregs to favor the development of an efficient antitumor immune response.

## 2. Chemokines: A Key Role in the Active Recruitment of Tregs in Tumors

The large chemokine family comprises about 50 small, secreted proteins structurally similar to cytokines and divided into four classes (CC, CXC, C and CX3C) that regulate cell trafficking. These proteins interact with a family of about twenty-seven transmembrane G protein-coupled receptors (GPCR) [50]. 

In tumors, the chemokine/chemokine receptor interactions play an important role in supporting tumor development, in part by the mobilization of Tregs. Moreover, the gain of expression by Tregs of the transcription factors and chemokine receptors associated with T-helper subsets may favor their ability to travel to specific peripheral sites to suppress their effector T-cell counterparts [51]. Among the different chemokine receptors, the role of several CC or CXC chemokines and their cognate receptors have been involved in the recruitment of Tregs in the TME.

### 2.1. Major Chemokine Receptors and Ligands Involved in Tregs Recruitment in the TME

#### 2.1.1. CCR4 and Its Cognate Ligands

**Figure 1:**
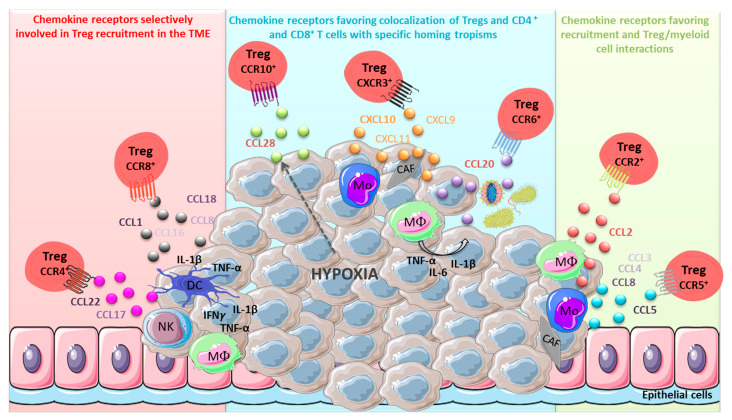
Chemokine/chemokine receptors axes involved in the recruitment of Tregs in the TME and the major mechanisms involved in the production of these chemokines in the TME. Several chemokine/chemokine receptors contribute to Treg recruitment at a tumor site according to their specific contexts; however, CCR4 and CCR8 appear as the most selectively expressed in TA-Tregs. NK: natural killer cells; MΦ: macrophages; Mo: monocytes; CAF: Cancer associated Fibroblasts.

Human CCR4 acts as the receptor for CCL17 and CCL22 and is expressed by Th2, Th17 and Th22 CD4^+^ T cells [52,53,54], as well as by Tregs [55] in the periphery (Figure 1). The enhanced expression of CCR4 by Tregs is implicated in their migration toward several non-lymphoid organs [56]. 

In addition, murine *Ccr4*-deficient Tregs are unable to infiltrate inflamed tissue and fail to control the immune responses in various models of inflammatory diseases [56]. CCR4 plays a major role in the recruitment of Tregs to human inflamed liver to limit the inflammatory responses [57]. In the tumor context, bone marrow chimera experiments in a model of murine glioma demonstrated the major role of CCR4 in the recruitment of Tregs in the brain TME to inhibit the antitumor immune response [58]. 

**Figure 2:**
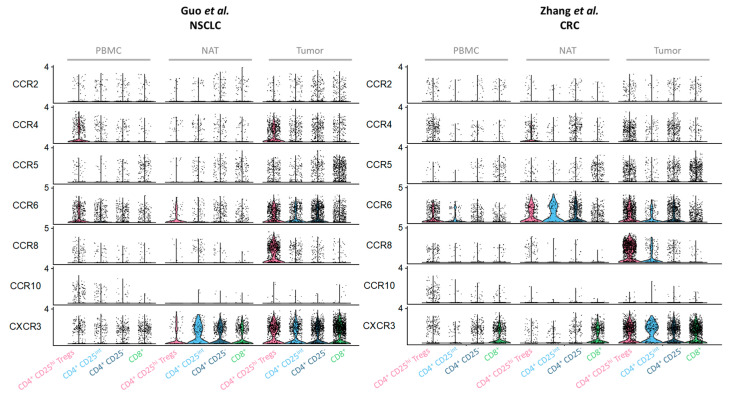
Evaluation of the chemokine receptor expression in human T-cell subsets in non-small cell lung cancer (NSCLC) and colorectal carcinoma (CRC) single-cell RNA sequencing (scRNA-seq) public datasets. Public scRNA-seq datasets of T cells issued from blood (PBMC), normal adjacent tissue (NAT) and tumor tissues of NSCLC patients (GSE 99254 and SMARTSeq2) [44] and CRC patients (GSE 108989 and SMARTSeq2) [65] were reanalyzed using bioinformatic tools based on the same clusters as described by the authors (pink: Tregs, sky blue: activated CD4^+^ Teff, dark blue: nonactivated CD4^+^ T cells and green: CD8^+^ T cells).

Furthermore, numerous reports have highlighted the role of CCR4 in the recruitment of Tregs in human tumors, such as breast, ovarian, colon, gastric, lung, brain and prostate carcinoma, as well as in melanoma and HL and diffuse large B-cell lymphoma [21,26,31,59,60,61,62,63,64]. The reanalysis of T-cell scRNA-seq datasets in NSCLC [44] and CRC [65] highlighted a higher *CCR4* gene expression mainly in Tregs in peripheral blood mononuclear cells (PBMC), whereas, in the TME, the *CCR4* gene is highly expressed in TA-Tregs but, also, upregulated on other T-cell subsets (Figure 2). 

CCL22, the dominant CCR4 ligand for Treg recruitment in tumors, has been described in different solid tumors (breast and cervical cancers, glioblastoma, squamous cell carcinoma (SCC), CRC and PDAC), where its expression correlates with Treg infiltrate and poor prognosis [22,25,31,46,66,67,68,69,70]. However, in several tumors, including gastric carcinoma, CCL17 has also been reported to participate in CCR4^+^ Treg chemoattraction [71].

CCL22 is produced by tumor-associated macrophages (TAM) and DC in CRC and PDAC [70], cervical carcinoma [25], HNSCC [66,72] and ovarian carcinoma [22]. The production of CCL22 is induced by inflammatory soluble mediators present in the TME, such as tumor cell-derived IL-1α in CRC and PDAC [70]. Interestingly, scRNA-seq studies focusing on immune cells infiltrating HCC reveal, through analyses of predicted receptor/ligand interactions, the importance of CCL22 secreted by DC to interact with CCR4^+^ Tregs [47]. Finally, CCL22 is also produced by primary cancer cells in breast, ovarian and HNSCC tumors [22,59,72,73] but not cancer cell lines, suggesting a major role of tumor environmental factors. We contribute to highlight the cooperation between TAM and NK cells through the secretion of IL-1β, TNF-α and IFN-γ, leading to the production of CCL22 by cancer cells [59]. In addition, IL-1β produced by cancer-associated fibroblasts (CAF) has also been described to favor the production of CCL22 by cancer cells in HNSCC [72,73]. Moreover, the microbiota has been suggested to favor CCL22 secretion by cancer cells in CRC [74].

#### 2.1.2. CCR8 and Its Cognate Ligands

**Figure 3:**
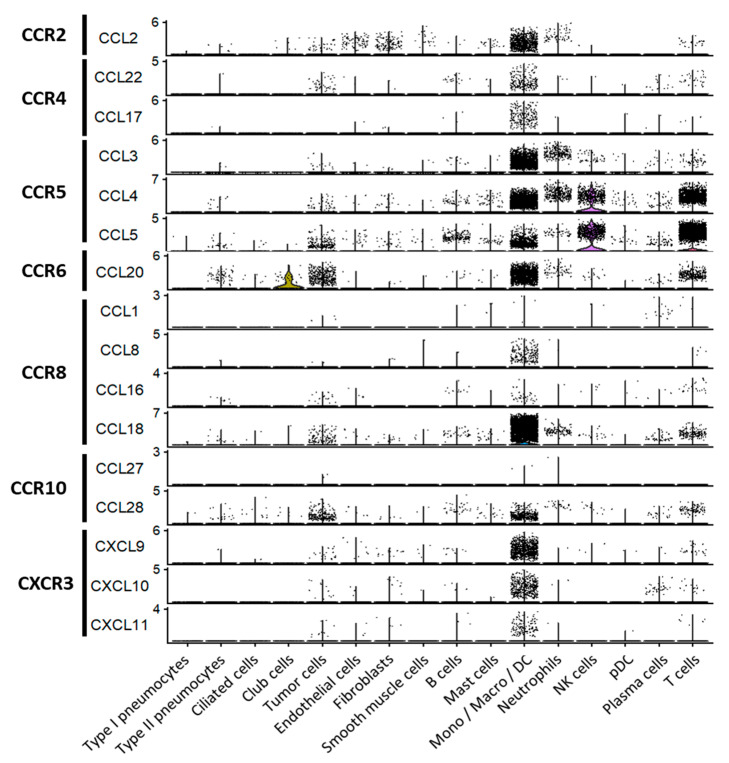
Characterization of cells (immune and nonimmune populations) expressing genes of chemokines in human NSCLC.

Human CCR8, which binds to CCL1, CCL8, CCL16 and CCL18, is expressed in blood by a subset of memory CD4^+^ T cells enriched in Th2 cells [75] and a subset of Tregs [76]. The role of CCR8 in Tregs physiopathology was initially demonstrated in the field of transplantation where *Ccr8*-deficient Tregs were unable to prevent T cell-induced graft-versus-host disease in lung and colon tissues [77]. Data from human tumor RNA-seq or scRNA-seq studies have demonstrated a high overexpression of the *CCR8* gene on TA-Tregs compared to Tregs from NAT and blood [43,46,78,79,80,81]. We confirmed this observation by reanalyzing scRNA-seq datasets of T cells in NSCLC [44] and CRC [65] (Figure 3). At the protein level, and in contrast to CCR4, CCR8 is almost exclusively expressed by TA-Tregs among TILs in different solid tumors (breast, NSCLC, CRC and MIBC [43,46,82]). Phenotypic characterization of Tregs in human NSCLC highlighted that CCR8^+^ Tregs also highly express costimulatory molecules (ICOS, OX40 and 4-1BB) and the transcription factors IRF4 and Helios, as well as Ki67, which mark effector Tregs functions. In accordance, CCR8 is upregulated upon TCR engagement on memory CD4^+^ T cells [83]. Furthermore, their presence is highly correlated with multiple exhaustion traits of effector CD4^+^ and CD8^+^ T cells [84]. In breast and CRC, a high tumor infiltration by CCR8^+^FOXP3^+^ Tregs correlates with a significantly shorter patient overall survival (OS) [43,46]. A similar observation has been reported in MIBC patients in whom the presence of CCR8^+^FOXP3^+^ Tregs is also associated with a reduced responsiveness to chemotherapy [82]. 

We reanalyzed the public scRNA-seq dataset (GSE 127465) from Zilionis et al. [85] to investigate, using bioinformatic tools, the cells able to produce the chemokines favoring Treg recruitment in the TME based on the clusters of cells identified by the authors.

Among CCR8 ligands, *CCL18* and, at lower levels, *CCL1* genes have been shown to be expressed by myeloid cells in the breast TME [46]. The reanalysis of the NSCLC scRNA-seq dataset [85] highlighted that *CCL1* and *CCL16* genes are very poorly detected in the TME, whereas *CCL8* and *CCL18* mRNA are well-detected. The *CCL8* gene is only detected on monocytes/TAM and the DC cluster, whereas the *CCL18* gene is expressed more widely on immune clusters (neutrophils, B cells, plasma cells and T cells) but, also, specifically, on tumor cell clusters from the lung tissue (Figure 3). The *CCL18* gene is also expressed at high levels by M2-type TAM infiltrating solid tumors [86] and, also, at lower levels by CAF [87] and cancer cells [88]. The mechanism of induction of these chemokines has not been described but could rely on inflammatory cytokines (IL-1β and TNF-α) present in the TME (Figure 1), as they have previously been reported to favor CCL1 secretion by DC in atopic skin inflammation [89].

In humans, CCL18 has been demonstrated to induce the migration of blood nTregs [90]. CCL18 is associated with a poor prognosis in solid tumors, but this is rather related to its role in tumor angiogenesis [91] than a role in Treg recruitment. Indeed, only one report, in a murine CRC model, suggests that CCL18 participates to Treg recruitment in the TME [92]. Another one reported, in the humanized breast tumor model, the ability of CCL18 to recruit naive CD4^+^ T cells and favor their differentiation into iTregs in the TME [93]. 

In contrast to other CCR8 ligands, the engagement of CCR8 by CCL1 on human Tregs has been reported to play a major role in their suppressive function. Indeed, the treatment of Tregs with CCL1, but not with CCL18, induces a STAT3-dependent increase of FOXP3, CD39, IL-10 and granzyme B, resulting in enhanced stability and immunosuppressive functions [94].

Moreover, CCL1 increases CCR8 expression on human Tregs, boosting their CCL1 sensitivity [94]. In this context, in murine CRC tumor models, targeting CCR8^+^ Tregs through either anti-CCL1 neutralizing monoclonal antibody (mAb) [95] or anti-CCR8 mAb [96] drastically reduced the tumor infiltration by Tregs while robustly enhancing the antitumor immune response. However, since in human tumors, CCL1 is only weakly detected in the TME (Figure 3), it would be important to investigate the role of anti-CCL18 or anti-CCL8-neutralizing mAbs or to target directly CCR8 on TA-Tregs. In this context, the ex vivo culture of cells from human MIBC tumors with anti-CCR8 mAb leads to a loss of FOXP3 expression in Tregs, favoring their destabilization and their trans-differentiation into IL-17A-producing cells [82]. Furthermore, in patients with RCC and other urothelial carcinoma, CCR8 is also detected, in addition to TA-Tregs, on M2-type TAM. These CCR8^+^ TAM that display activated STAT3 are capable to increase the FOXP3 expression in infiltrating CD4^+^ T cells, suggesting the generation of iTregs that will favor tumor progression [97].

### 2.2. Chemokine/Chemokine Receptors Favoring Recruitment and Treg/Myeloid Cell Interactions

#### 2.2.1. CCR2/CCL2

Even if the CCR2 axis is dominantly involved in TAM and MDSC recruitment at tumor sites, it also contributes to Treg recruitment in the TME, although, in human tumors, the expression of CCR2 on T cells does not appear selective for TA-Tregs, at least in NSCLC [44] and CRC [65] (Figure 2). CCL2, one of the CCR2 ligands, is a potent chemoattractant of different immune cells, including monocytes/macrophages [98,99] and myeloid-derived suppressor cells (MDSC) [58,100] but, also, a subset of Tregs that exhibit high suppressive functions [101]. In a murine model of glioma, CD163^+^ TAM and microglial cells within the TME are responsible for CCL2 secretion (Figure 1), recruiting Tregs and MDSC-mediating immunosuppression [58]. Furthermore, in fibrosarcoma (MCA) and mammary (PyMT) murine tumor models, the CCR2/CCL2 axis participates in the recruitment of Tregs from the lymph node to the tumor, as *Ccr2*-deficient Tregs were unable to infiltrate the TME [101]. In humans, CCL2 and CCR2 expression strongly correlates with poor patient prognosis [102,103] and a role for CCR2^+^ Tregs in this deleterious impact cannot be excluded. Finally, in HNSCC, tumor progression has been associated with an increased detection of CCR2^+^ TA-Tregs [101].

#### 2.2.2. CCR5/CCL5

As for the CCR2 axis, the CCR5 axis is not only related to Treg recruitment, because its ligands (CCL3, CCL4, CCL5 and CCL8 [104]) are also involved in tumor progression and metastases. In the TME, CCL5 could be produced by cancer cells, endothelial cells and TAM, as well as CAF (Figure 1) (for review, [105]), and CCR5 is expressed on a range of immune cells, including TAM, DC and T cells [106]. However, in human CRC, higher levels of CCR5 have been detected on TA-Tregs compared to effector CD4^+^ T cells and to Tregs from distal colon tissue [107] (also shown in Figure 2). Interestingly, ex vivo, these CCR5^+^ TA-Tregs are more potent suppressors than their CCR5^neg^ counterparts [107], suggesting that CCR5 expression not only favors Treg recruitment to the tumor but, also, defines a subset with more potent suppressive functions. Moreover, in murine DMBA-induced SCC, the CCR5-dependent homing of Tregs in the TME was demonstrated to contribute to tumor development [108]. Consistently, in mice with *Ccr5*-deficient Tregs, tumors are less infiltrated by Tregs, and tumor growth is delayed [109]. Finally, in a murine model of PDAC, the disruption of CCR5/CCL5 signaling, either by reducing CCL5 production by tumor cells (shRNA) or by systemic administration of the CCR5 inhibitor TAK-779, results in decreased Treg recruitment to the tumor and smaller tumors [110] that led the authors to suggest the potential use of such an inhibitor in clinic to inhibit Treg recruitment. However, as CCL5 could also favor the recruitment of antitumor effectors such as NK cells, Th1 CD4^+^ T cells and cytotoxic CD8^+^ T lymphocytes (for review, [111]), its blockade will be deleterious, except if combined in a bispecific format with a specific TA-Treg marker.

Both CCR2/CCR2L and CCR5/CCR5L may favor recruitement at the same site of Tregs and TAM/MDSC, favoring their tight interactions and strenghtening immuno-suppressive networks.

### 2.3. CCR6, CCR10 and CXCR3: Receptors Favoring Co-Localisation of Tregs with CD4^+^ or CD8^+^ Effector T Cells with Specific Homing Tropisms

#### 2.3.1. CCR6/CCL20

CCR6 was identified to be the sole receptor for CCL20 [112]. CCR6 is expressed by numerous leukocyte subsets, such as memory CD4^+^ T cells, B cells, immature DC, NKT cells, type-3 innate lymphoid cells (ILC3) and neutrophils. In blood and tissues, CD4^+^ T cells expressing CCR6 belong to Th17, Th1.17 and Th22 and a subpopulation of Tregs [52,113]. CCR6 plays a major role in the specific migration of these cells toward inflammation sites, as CCL20 is upregulated by proinflammatory mediators and is prominently expressed in the mucosal tissue (gut and lungs), liver and thymus [114].

CCL20 is upregulated in the TME of human solid tumors (breast, ovarian, cervix, liver, prostate, pancreas and colon) (for review, [115]. CCL20 is produced by TAM, as well as tumor and stromal cells (Figure 1). Indeed, in NSCLC, the analysis of the scRNA-seq dataset generated by Zilionis et al. [85] highlighted *CCL20* gene expression in the mono/TAM/DC cluster and, to a lesser extent, in the T cell cluster but, also, in tumor cells and specific lung epithelial cell (club cells and pneumocytes) clusters (Figure 3). Nevertheless, a recent study in CRC showed that cancer cells alone do not secrete CCL20 but require environmental factors for its production [74], suggesting that other populations cooperate with cancer cells for CCL20 synthesis. TAM favors CCL20 production by cancer cells through their secretion of TNF-α, IL-1β and IL-6. Moreover, the depletion of TAM in a murine CRC model strongly decreased CCL20 production, leading to tumor growth inhibition [116]. In addition, the composition of the gut microbiota, by triggering Toll-like receptor (TLR) signaling in cancer cells, can promote their CCL20 production [74].

CCR6^+^ Tregs are enriched in human solid tumors such as NSCLC, HNSCC, laryngeal and esophageal SCC and chemotherapy-resistant CRC compared to NAT and PBMC (see, also Figure 2) and are associated with tumor progression [117,118,119,120,121]. These CCR6^+^ TA-Tregs present a higher suppressive activity [119] that increases with advanced tumor stages. This infiltration could rely on the inflammatory context of these tumors favoring CCL20 production, but the exact role of the CCR6/CCL20 axis on Treg migration to the tumors remains poorly investigated. Moreover, CCL20 is associated with a poor prognosis and resistance to chemotherapy (for review, [115]) and has recently been reported as enhancing the self-renewal and stemness of cancer stem cells [122]. In breast carcinoma, CCL20 has also been associated with the epithelial-to-mesenchymal transition [123] that may participate in cancer cell invasiveness/metastasis. Moreover, in human tumors (esophageal SCC [124], NSCLC [125] and HNSCC [126]), CCL20 favors the recruitment of Th17 CD4^+^ T cells that also participate in tumor progression. Altogether, these results argue in favor of a CCR6 blockade to inhibit tumor progression.

#### 2.3.2. CCR10 and Its Ligands

CCR10 is expressed by Th22 and B lymphocytes, allowing their recruitment in either mucosal or cutaneous epithelial sites. Mucosal homing through CCR10 engagement is driven by the ligand CCL28, which is expressed by columnar epithelia in the gut, lung, breast and salivary glands [127], whereas homing to the skin is triggered by CCL27 [128], the alternative CCR10 ligand. In the liver, CCL28, produced by epithelial cells during chronic inflammation, favors the recruitment of CCR10^+^ Tregs to limit tissue damage [129]. Even though very few data describe the CCR10 expression by TA-Tregs in the TME, several analyses reported the role of CCL28 on Treg accumulation in tumors. In a murine model of ovarian cancer, the overexpression of CCL28 by tumor cells (Figure 1) favors a preferential recruitment of CCR10^+^ Tregs, resulting in an accelerated tumor growth [130]. In NSCLC, the reanalysis of the scRNA-seq tumor dataset from Zilionis et al. [85] allowed us to show that *CCL28* gene is detected within clusters of tumor cells and mono/TAM/DC, whereas the *CCL27* gene is not detected (Figure 3). However, the *CCR10* gene is not detected in TA-Tregs in both NSCLC [44] and CRC [65] scRNA-seq T-cell datasets, whereas it is present in a small subset of Tregs in the periphery (PBMC) (Figure 2). This suggests that CCL28/CCR10 is not involved in the recruitment of Tregs in the TME in these tumors.

In contrast, in human ovarian and liver tumors, hypoxia favors the production of high CCL28 levels by tumor cells (Figure 1) that are correlated with poor patient survival [130] and with the recruitment of CCR10-expressing Tregs in the TME [130,131]. This recruitment of CCR10^+^ Tregs in a hypoxic environment might have an increased immunosuppressive capacity [132,133]. However, this phenomenon could depend on the tumor type. Indeed, in basal-like breast carcinoma [134], NSCLC [135] and advanced cervical cancers [136], the recruitment of Tregs in hypoxic TME was associated with CXCR4 and CXCL12 expression, while the role of the CCL28/CCR10 axis was not investigated so far.

#### 2.3.3. CXCR3 and Its Ligands

CXCR3 is expressed in immune cells such as monocytes, DC, NK and NKT cells, CD8^+^ T cells, Th1 and Th1.17 cells but, also, some cancer cells (for review, [137,138]). CXCR3 is known to have a key role in T-cell trafficking to inflammatory sites and is required for effector cell trafficking to tumors [139,140]. CXCR3 exerts its biological effects through engagement by three IFN-γ inducible ligands (CXCL9, CXCL10 and CXCL11) that are mainly secreted by monocytes, endothelial cells, fibroblasts and tumor cells (Figure 1). CXCL10 and CXCL11 could also be induced in response to type-I (IFN-α and IFN-β) and type-III IFN (IFN-λ) [141].

The Campbell’s group first described in mice a subset of FOXP3^+^ Tregs expressing CXCR3 and the transcription factor T-bet [142]. This population is highly expanded during type-I inflammation and exhibits homeostatic and migratory properties optimized for the suppression of Th1 responses in vivo. The existence of CXCR3^+^ T-bet^+^ FOXP3^+^ Tregs has been confirmed in humans, both in healthy individuals and in patients with autoimmune diseases [143,144]. Importantly, a recent study in mice demonstrated, by the deletion of *Tbx21* (coding for T-bet) in FOXP3^+^ Tregs, the major role of CXCR3 in the migration of Tregs to the inflammatory tissue in close vicinity to effector CD8^+^ T cells. These CXCR3^+^ Tregs, by producing active TGF-β, promote in situ the differentiation of effector CD8^+^ T cells into resident memory T cells (T_RM_) [145] that play a role in the antitumor immune response [146]. The reanalysis of both NSCLC [44] and CRC [65] scRNA-seq T-cell dataset highlights that, in addition to its expression on conventional CD8^+^ and CD4^+^ T cells, the *CXCR3* gene is expressed at a higher level on Tregs in the TME compared to NAT and PBMC (Figure 2), suggesting a specific recruitment in the TME. This is in line with the detection in the NSCLC scRNA-seq dataset from Zilionis et al. [85] of all the CXCR3 ligands in the mono/TAM/DC cluster (Figure 3), *CXCL9* and *CXCL10* genes being detected at the highest intensity. Moreover, a high enrichment in CXCR3^+^ TA-Tregs co-expressing T-bet was described in human ovarian carcinoma, particularly in the solid tumor mass [147]. These CXCR3^+.^ TA-Tregs co-express Helios, suggesting that they originate from nTregs and suppress the proliferation and IFN-γ secretion by effector CD4^+^ T cells. Of interest, the authors reported a correlation between the proportion of CXCR3^+^ TA-Tregs and those of CXCR3^+^ TA-effector CD4^+^ T cells, consistent with the expression of CXCR3 ligands. This pro-tumoral effect of CXCR3^+^ Tregs was also observed in HCC, where a correlation could be made between the infiltration of CXCR3^+^ Tregs in the TME induced by CXCL10 and increased tumor growth and HCC recurrence after liver transplantation [148]. Moreover, in a chemically induced murine model of skin carcinoma, *Cxcr3*-deficient mice developed fewer tumors compared to wild-type (WT) mice. This observation was linked to a reduced presence of CXCR3^+^ T cells, suggesting a pro-tumoral activity of these TILs that could rely on CXCR3^+^ Tregs [149].

#### 2.3.4. Conclusions

All these results demonstrate that different chemokine receptor/ligand pairs contribute to Treg recruitment at the tumor site, according to specific contexts; however, CCR4 and CCR8 appear as the most selectively expressed on TA-Tregs compared to Tregs from the periphery or the healthy tissues (Figure 2). The targeting of CCR4 and CCR8 will be helpful to specifically deplete TA-Tregs or prevent their recruitment in the TME to avoid systemic or tissue-specific autoimmune manifestations. Moreover, the specific targeting of chemokine receptors only expressed by TA-Tregs will help to maintain the recruitment of effector T cells in the TME to favor antitumor immunity.

## 3. Expansion in the Tumor Environment

We and others reported an intra-tumoral Treg proliferation in different solid tumors [21,27,46,107,150]. Different mechanisms have been described to explain this in situ proliferation, including the role of the ICOS/ICOS-L axis, the importance of the TNF receptor superfamily members and a potential role of metabolic perturbations in the TME.

### 3.1. Role of ICOS-ICOS-L

ICOS, a T-cell costimulatory molecule of the CTLA-4/PD-1/CD28 family, plays a nonoverlapping function with CD28 [151] and is highly expressed by TA-Tregs in breast carcinoma [21,152], ovarian carcinoma [153,154], follicular lymphoma [155], melanoma [156,157], RCC [158] and gastric cancer [159]. In addition, recent scRNA-seq studies have confirmed that ICOS participates in the gene signature of effector TA-Tregs in the TME [43,44,45,47,78,81,160]. In addition, the detection of ICOS^+^ TA-Tregs in solid tumors is associated with a poor patient prognosis [152,153,158,159].

**Figure 4:**
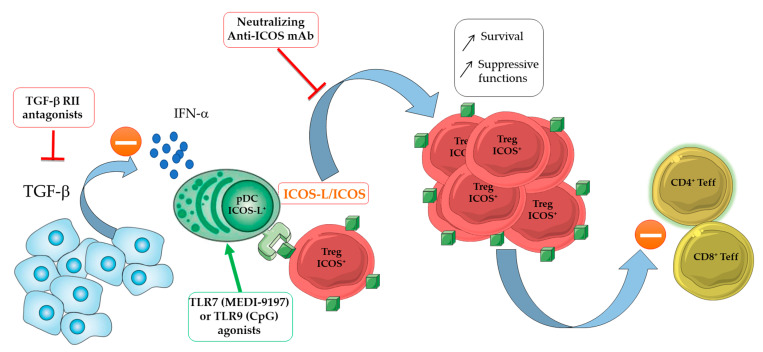
The TA-plasmacytoid DC (pDC)/Treg interaction is at the center of the immunosuppressive network in the breast TME. pDC capacity to secrete type-I IFN (IFN-α) is altered by TGF-β produced in the TME. The interaction between pDC and Tregs through ICOS/ICOS-L favors the expansion and survival of Tregs that is amplified by the absence of type-I IFN. These TA-Tregs inhibit the functionality of tumor-infiltrating CD4^+^ and CD8^+^ Teff. Therapeutic strategies with TGFβ-RII antagonists, neutralizing anti-hICOS mAbs, TLR7 or TLR9 agonists or their combination could reduce this immunosuppressive network.

Despite their in situ expression of Ki67 in the TME [46,152,153,161], TA-Tregs do not proliferate in vitro under TCR stimulation with agonist anti-CD3/anti-CD28 mAb-coated beads and exogenous IL-2 but require a signal through ICOS engagement [152]. In this context, ICOS-L is expressed either by TA-plasmacytoid DC (pDC) in breast [152], ovarian [153] and gastric [162] cancers or by tumor cells in follicular lymphoma [155], acute myeloid leukemia [163] or melanoma [156] and plays a major role in the expansion of ICOS^+^ TA-Tregs (Figure 4).

Indeed, their proliferation is completely blocked by the addition of antagonist mAbs directed against ICOS or ICOS-L. In addition, in a humanized mouse model of breast carcinoma, the addition of a neutralizing anti-ICOS mAb reduced the Treg proportion and improved the CD4^+^ T cell proliferation [164].

Similar observations were made for the differentiation of Tregs in a physiological context [165]. Apart from its role in Treg proliferation, ICOS engagement by ICOS-L favors Treg survival and suppressive functions. Actually, one underlying mechanism relies on NFAT activation, which boosts the transcription activity of FOXP3 that, in turns, enables the expression of target genes. In addition, ICOS signaling activates the antiapoptotic AKT signaling pathway, leading to an increased Tregs survival [166]. *Icos* deficiency in mice is associated with CNS2 hypermethylation and a loss of FOXP3 expression, as well as a reduction of 30% in the Treg pool [167]. Finally, in mice, anti-ICOS blocking mAb disrupts Treg activity [168,169].

### 3.2. Role of TNFR2

TNF-α binds to two TNF receptors. TNFR1 belongs to the death receptor family characterized by a cytoplasmic death domain (DD), which enables the triggering of cytotoxic signaling [170]. TNFR2 is highly expressed on murine and human Tregs and is a member of the TNF receptor-associated factor (TRAF)-interacting receptor family. It lacks a DD and directly interacts with TRAF family members (TRAF1, TRAF2 and cIAP) [171]. In contrast with the TNFR1-mediated negative effects on Tregs [172], TNFR2 actively participated to the maintenance of their proliferation [173,174] and their suppressive functions [175]. In this context, therapeutic strategies targeting TNFR2 through agonists have been developed to expand Tregs to treat autoimmune diseases [176,177,178]. As all anti-hTNFR2 antagonists have been designed to eliminate TNFR2-expressing cells (tumor cells or Tregs) by antibody-dependent cellular cytotoxicity (ADCC), they will not help to decipher the impact of TNFR2 inhibition in human Tregs. In this objective, Atretkhany et al. developed a doubly humanized *TNF/TNFR2* mouse line with a conditional inactivation of the human *TNFR2* gene in FOXP3^+^ Tregs [179]. The inactivation of *TNFR2* downregulated the expression of the Treg signature molecules (Foxp3, CD25, CTLA-4 and GITR) and reduced the Treg suppressive function in vitro [179]. In this humanized *TNF/TNFR2* model, the deficiency of *TNFR2* restricted to Tregs led to significant exacerbation of experimental autoimmune encephalomyelitis, accompanied by a reduced capacity to control the Th17-mediated immune response. The manuscript from Laine A et al., in the same issue, will detail the role of TNFR2 in Treg suppressive functions.

In tumor context, the scRNA-seq analysis of TILs highlighted the upregulation of TNFR2 on TA-Tregs compared to other TA-T cell subsets, except for some TA-CD8^+^ T_RM_ [45,49]. At the protein level, TNFR2 is upregulated in the TME compared to patient blood in different cancers, such as in NSCLC [180], cervical cancer [181] and HCC [182], as well as in metastatic ovarian carcinoma [183]. These TNFR2^+^ Tregs exhibit strong suppressive functions [184] and are associated with a high tumor grade and with a poor prognosis [180,185]. Moreover, TNF-α produced by stromal cells could participate in the expansion and suppressive function of TA-Tregs. Indeed, the treatment of melanoma-bearing mice with TNF-α induced the expansion of TA-Tregs [186]. Moreover, in a murine model of CRC (CT26), CD103-expressing effector TA-Tregs express higher levels of TNFR2, as observed in human tumors, and the blockade of the TNFR2/TNF-α axis with a sTNFR2-Fc chimera efficiently inhibits their TNF-α-induced expansion in vitro [187]. In addition, the in vivo treatment with a sTNFR2-Fc chimera abolished the TA-Treg expansion in CRC and HCC mice tumor models [187]. Furthermore, in human ovarian carcinoma and cutaneous T-cell lymphoma (CTCL and Sezary syndrome), the TNFR2 antagonist reduced the ovarian carcinoma development by inducing both cancer cell death and TA-Tregs depletion [188,189] but has little inhibitory effects on blood Tregs or Tregs from healthy donors, likely due to their lower expression of TNFR2.

### 3.3. Role of Other TNF Receptor Superfamily Costimulatory Molecules (4-1BB, OX40 and GITR)

Human and murine TA-Tregs express other members of the TNF receptor superfamily (OX40, 4-1BB and GITR) with a costimulatory capacity that could modulate their proliferation [43]. However, the expression pattern of some of these receptors remains highly different between humans and mice. Whereas these molecules are expressed at the basal level on Tregs in mice [190,191,192,193], their expression is inducible on human Treg subsets upon activation. Indeed, in vivo inflammatory conditions in humans, such as inflamed joints from juvenile idiopathic arthritis [194] or in vitro TCR stimulation [190,195,196], upregulate GITR, OX40 and 4-1BB expression on Tregs but, also, on effector CD4^+^ T cells.

Nevertheless, even if the observations about the role of these receptors on Tregs in vivo are difficult to interpret due to the use of mAbs that deplete Tregs rather than blocking their function (i.e., DTA-1 clone for GITR), some data can still help in the comprehension of the implication of these receptors. In vitro co-stimulation with the GITR ligand (GITR-L), when combined with TCR signaling, increases murine nTreg precursor differentiation and synergizes with IL-2-induced STAT5 signaling to increase their maturation and proliferation [197,198,199,200]. Moreover, pentameric GITR-L, mimicking the natural GITR-L [201], induces the proliferation of murine Tregs in vitro and in vivo [198]. In *Gitrl*-transgenic mice, GITR stimulation leads to Treg amplification and stimulates their acquisition of an effector phenotype without repressing their suppressive function [202]. However, the discrepancies that exist between humans and mice for the expression pattern of GITR [192] could explain the contradictory results. Indeed, in humans, the use of agonist anti-GITR mAb (MK-4166 and hIgG1) in vitro inhibits the generation of iTregs but does not alter the differentiated Treg population. The addition of MK-4166 favors a decreased expression of FOXP3 that could explain the partial reduced suppressive function of Tregs in vitro when using the agonist mAb [192]. Nevertheless, the first clinical evaluation of the fully human anti-GITR agonist from Bristol-Meyers Squibb(BMS) (New York, NY, USA), (BMS-986156, hIgG1) in phase 1 alone or combined with anti-PD-1 in patients with advanced solid tumors did not report consistent and significant modulation in TA-Tregs or CD8^+^ T cells [203].

OX40 is highly expressed on TILs, especially on Tregs, in many cancers, including melanoma, CRC, HNSCC, breast cancer and B-cell lymphoma [204], and used as a marker for Ag-specific TA-Tregs [205]. In addition to favor effector CD4^+^ and CD8^+^ T cell expansion, agonist rat IgG1 anti-OX40 mAb has also been shown to deplete murine TA-Tregs, expressing higher levels of OX40 by ADCC caused by FcγR-expressing myeloid and NK cells present within the TME [206], which resulted in an altered Teff/Treg cell ratio in tumors. OX40-L is expressed by liver resident monocytes and macrophages in HCV-derived HCC [207] but, also, by CAF in NSCLC after cisplatin-based chemotherapy [208]. The triggering of OX40 on Tregs with OX40-L hexamer fusion protein reduces their suppressive function in vitro and in vivo in mice [209] and in vitro in humans [210].

The expression of 4-1BB (CD137) functions as a costimulatory molecule that potentiates TCR-mediated NF-κB signaling, leading to the increased activation and proliferation of T and NK cells. 4-1BB has been previously reported as a marker of Ag-specific Tregs [211] and is also associated with a highly activated and functional TA-Treg population in the TME [44]. In vitro, the impact of 4-1BB co-stimulation on Treg expansion is still debated. Some studies reported an enhanced expansion of Tregs [212,213], whereas others only showed an inhibition of their suppressive capacity [214] or a decreased conversion of effector CD4^+^ T cells into iTregs after 4-1BB stimulation [215]. Thus, the functional consequences of 4-1BB engagement on Tregs remain contentious, and further studies are required to clarify the impact of agonist anti-4-1BB mAbs or synthetic 4-1BB ligands on human Treg expansion and suppressive capacity. Such knowledge would be essential in the context of immunotherapy strategies aiming to activate effector CD4^+^ or CD8^+^ T cells.

### 3.4. Negative Regulators

In addition to the expression of activating immune checkpoints (ICP) on the TA-Tregs described above, the RNA-seq or scRNA-seq studies dedicated to T cells [43,44,79] highlighted their increased expression of inhibitory ICP genes, such as *CTLA-4*, *TIGIT* and *HAVCR2* (coding for TIM-3), compared to both Tregs from peripheral blood or NAT. These data were confirmed at the protein level in adequacy with an effector phenotype and higher suppressive function [41,46,216,217]. In contrast, the expression of PD-1 is less clear. At the mRNA level, *PDCD1* mRNA could be either overexpressed or not on TA-Tregs [44,46,218] or restricted to specific clusters [160]. However, at the protein level, PD-1 is overexpressed on TA-Tregs in breast cancers (BC) [49,219], ovarian carcinoma [154], NSCLC, gastric cancer and CRC [220], as well as in more advanced carcinoma [221]. This discrepancy could result from the limits of scRNA-seq strategies (10X Genomics and InDrop).

### 3.5. Metabolism

Where effector CD4^+^ and CD8^+^ T cells mainly use aerobic glycolysis and anabolism to support bioenergetic demands, Tregs rely on fatty acid oxidation (FAO) and oxidative phosphorylation (OXPHOS) to support their differentiation and function [222,223]. This results in part from the inhibition of c-MYC by FOXP3, which makes Tregs shift from glycolysis to OXPHOS. In this context, deprivation in glucose, induced by the Warburg effect in the TME, is detrimental to effector T cells but only minimally impacts TA-Tregs. However, glycolysis remains important for Treg functions. Procaccini C. et al. have reported the activation of glycolysis in Tregs to support their proliferation [224]. Moreover, Kishore et al. have also shown that the migration of Tregs to inflammatory sites requires glycolysis, and this is linked to glucokinase induction by PI3K-mTORC2 [225].

In addition, lactate, which is the major metabolite resulting from a glycolytic switch, is abundant in the TME and could enhance the ability of TA-pDC to favor Treg proliferation by either reducing their capacity to produce IFN-α upon TLR stimulation or favoring their production of kynurenine from tryptophan through IDO activity [226]. Furthermore, the mitochondrial respiratory chain is required for the suppressive function, stability and survival of Tregs [227]. In mice, CD36, a scavenger receptor, plays a central role as a metabolic modulator. Indeed, CD36, responsible for long-chain free fatty acid (FFA) and oxidized low-density lipoprotein uptake, is selectively upregulated in TA-Tregs. CD36 finetunes the mitochondrial fitness and NAD levels to program Treg adaptation to a lactic acid-enriched TME [228] and favors Treg survival dependent on lipid oxidation [223,229,230]. In addition, in mice, the FFA, de novo synthetized from glucose by tumor cells, are taken up more efficiently by Tregs than other T cells [230], maybe due to their higher CD36 expression, reducing their apoptosis and favoring their in vivo expansion and suppressive function in murine gastric tumor models [231]. In 8% of gastric cancer patients, the oncogenic driver mutation in RHOA (Y42) in tumor cells leads to the activation of the PI3K/AKT/mTOR pathway and increases the production of FFA that favors Treg survival. In this context, the treatment of a murine model of gastric cancer harboring *RhoA-Y42* mutation, with anti-CD36 mAb or a selective PI3Kβ small molecule inhibitor (GSK2636771), inhibits AKT phosphorylation and strongly decreases the number of TA-Tregs by reducing the total FFA production in the TME [231]. However, in humans, a reanalysis of the scRNA-seq data failed to highlight the expression of CD36 on TA-Tregs, suggesting that CD36 has a divergent role between mice and humans but that other transporters, such as SLCA27A1, may play a role [232]. This discrepancy could also result from a low number of *CD36* transcripts not visualized by the current scRNA-seq strategies (10X Genomics and InDrop).

The characterization of metabolic pathways selectively involved in TA-Treg amplification in the TME will help to define attractive targets. The combination with other drugs reactivating the exhausted effector T cells will favor the restoration of an efficient antitumor immune response.

## 4. Treg Stabilization

The sustained expression of FOXP3 in Tregs is required for lineage commitment and stability. Several key mechanisms contribute to the regulation of FOXP3 expression, including cytokine signaling, epigenetic control with demethylation of CpG motifs in the TSDR [233] and interactions of FOXP3 with other proteins. Works in the autoimmune and inflammation fields have shown that, under highly inflammatory conditions, Tregs can become destabilized, lose the expression of FOXP3 and convert to effector CD4^+^ T cells [234,235,236]. In cancer, the opportunity to convert immune-suppressive TA-Tregs into antitumor effector CD4^+^ T cells remains an attractive therapeutic aim. This section will review several mechanisms associated with Treg stability.

### 4.1. Helios

The generation of mice with a tissue-specific deletion of Helios allowed to demonstrate that a loss of *Helios* in most T cells (CD4^Cre^-driven loss of *Helios*) did not affect their development, function or immune homeostasis [11]. Additionally, Cantor’s group [237] demonstrated that mice with *Helios* deficiency in Tregs did not present any alteration in Treg development, nor the negative selection of effector CD4^+^ T cells [237]. The deletion of *Helios* specifically in Tregs also did not result in a rapid onset of autoimmunity, unlike *Foxp3*-deficient mice (Scurfy) but to an autoimmune-like syndrome with a lymphocytic infiltration of immune cells in nonlymphoid tissues and auto-Ab production appearing after five months [237,238]. This demonstrates that the loss of Helios expression by Tregs induces defective Treg functions, leading to autoimmune disease development. Furthermore, after the vaccination of mice with sheep red blood cells, *Helios*-deficient Tregs develop an unstable phenotype with reduced Foxp3 expression and increase the effector cytokine expression (IFN-γ and IL-17) due to a reduced STAT5 pathway activation, suggesting their conversion into effector CD4^+^ T cells [237,238,239]. Thus, it appears that Helios is critical in maintaining Treg identity, repressing their ability to express effector cytokines. Moreover, in mouse models of melanoma (B16F10) and CRC (MC38), tumor development was reduced in mice with *Helios*-deficient Tregs due to an enhanced tumor immunity. The authors reported that the underlying mechanism was the conversion of Tregs with an unstable phenotype to IFN-γ- and TNF-α-producing Teff [239]. However, a role of an impaired suppressive function of Tregs due to *Helios* deficiency cannot be ruled out.

### 4.2. STAT-5

IL-2 signaling is considered as a major regulator for controlling the homeostasis and function of Tregs [240,241]. Unlike effector CD4^+^ T cells, Tregs exhibit a predominant activation of downstream STAT5 over the MAPK and PI3K pathways, partly due to their high expression of the PTEN phosphatase [241,242]. STAT5, by directly binding the CNS2 region of the *Foxp3* gene, regulates the inheritable stability of FOXP3 expression and suppressive functions of Tregs [243,244,245,246]. In mice, the serine-threonine kinase Mst1 (Hippo family) was identified as a signal-dependent amplifier of IL-2–STAT5 activity in Tregs [247]. Indeed, Mst1 regulates the Foxp3 expression and Treg development/function and inhibits autoimmunity through modulating Foxo1/3 stability [248]. Interestingly, patients with *MST1(STK4)*-null mutations exhibit immunodeficiency with autoimmune phenotypes [249,250], partly due to defaults in Treg development/function.

### 4.3. Von Hippel-Lindau E3-Ubiquitin Ligase

In murine models, the Von Hippel-Lindau (VHL) E3-ubiquitin ligase was highlighted to stabilize Tregs, as *Vhl*-deficient Tregs were not able to prevent colitis due to their conversion into Th1-like effector T cells with excessive IFN-γ secretion [251]. The most well-documented substrate of the VHL complex is hypoxia-inducible factor-1α (HIF-1α), an oxygen sensor and transcription factor that controls the expression of various genes responsible for angiogenesis and glucose metabolism under low oxygen levels [252]. The deletion of VHL expression selectively in Tregs using *Vhl*^fl/fl^ *FOXP3*^cre^ mice demonstrated that VHL plays an important role in maintaining Tregs stability and function via modulating the HIF-1α pathway [251]. More recently, other ubiquitin ligases in human (RNF31) [253] and murine models (Hrd1) [254] have been described to play a role in Treg stability in other contexts than cancer. Interestingly, a recent paper from Bluestone’s group [255] reported the development of a CRISPR-based pooled screening platform used to identify gene regulatory programs that promote or disrupt Foxp3 expression in mouse Tregs. The targeted loss-of-function screen of around 500 nuclear factors highlighted that Rnf20, another E3 ubiquitin ligase, can serve as a negative regulator of Foxp3. In contrast, they identified Usp22, a member of the de-ubiquitination module of the Spt-Ada-Gcn5-acetyltransferase (SAGA) chromatin-modifying complex, as a positive regulator stabilizing the Foxp3 expression. Usp22 acts at the transcriptional level by histone de-ubiquitination across the *Foxp3* locus, favoring an increased transcription of *Foxp3* but, also, at the post-transcriptional level by the de-ubiquitination of *FOXP3* that favors its stability. The knockdown of *USP22* on human Tregs significantly decreases the frequency and intensity of FOXP3 expression. They also confirmed the reduced Foxp3 expression on *Usp22*-deficient Tregs and its rescue by the ablation of Rnf20, revealing a reciprocal ubiquitin switch in murine Tregs. Moreover, an injection of tumor cells (EG7, B16 and LLC-1) in mice with *Usp22*-deficient Tregs reduced tumor development through the downregulation of the Treg suppressive functions and their abundance in the TME, consequently enhancing the antitumor immune response.

### 4.4. Neuropilin 1 (NRP1)

Ectopic expression and chromatin immunoprecipitation experiments demonstrated that the murine *Nrp1* gene is a direct target of Foxp3-mediated transcriptional regulation [256,257]. NRP1 co-expression with Helios was initially considered as a marker for nTregs in mice [258,259]. Unlike murine nTregs, human Tregs in blood do not constitutively express NRP1. However, in human lymph nodes, NRP1 identifies a discrete population of activated Tregs [260], indicating that immune processes may regulate its expression in vivo. Interestingly, NRP1^+^ Tregs are highly enriched in the TME of CRC [261], melanoma and HNSCC [262] and are associated with a poor prognosis in HNSCC [262]. In the context of cancer, NRP1 could participate in Tregs recruitment to the tumor by acting as a coreceptor for VEGF [263]. In addition, murine *Nrp1*-deficient Tregs are associated with a profound tumor resistance due to Treg functional fragility with the acquisition of characteristic T-helper lineage markers (such as T-bet, CXCR3, IRF4 and RORγt), even if they retain their Foxp3 expression [264,265]. This observation thus indicates that Nrp1 is important in the maintenance of TA-Treg stability and function. Mechanistically, upon TCR engagement, Nrp1 recruits PTEN to the immunological synapse, blocking the potentially toxic activation of AKT, which can inhibit FOXO activity and drives glycolytic metabolism, thus preserving Treg stability and function [262,264] (see Section 4 and Section 5).

### 4.5. PI3K/AKT/mTOR

Recent evidence showed that the altered PI3K/AKT pathway axis, downstream TCR, costimulatory signaling (ICOS and CD28) and IL-2 receptor play a critical role in the expression of FOXP3 and, subsequently, regulate the development and suppressive function of Tregs [266,267], as well as their metabolism [268,269]. However, contradictory results have been published concerning the role of PI3K on Treg differentiation and function. Through the mTORC2 complex, PI3K/AKT signaling induces the phosphorylation of the transcription factor FOXO1, a key regulator of Treg activity. This prevents its nucleus localization, leading to a reduced expression of several Treg signature genes (e.g., *Helios*), whereas the transcription factors associated with Th1 lineage (*Tbx21*) and inflammatory cytokines are upregulated. In contrast, the mTORC1 complex is important for coordinating Treg proliferation and CTLA-4/ICOS expression, contributing to Treg suppressive function partly through inhibiting the mTORC2 pathway [270]. The AKT/mTOR axis is widely acknowledged as a crucial negative regulator of Treg de novo differentiation [267,271,272] and population expansion [273]. Indeed, inhibition of the mTOR pathway is necessary for stable Treg lineage commitment, after which mTOR signals are finely tuned to support Treg function without compromising their stability. In addition, the specific ablation of *Pten*, the primary negative regulator of PI3K, in murine Tregs impairs the mitochondrial fitness, upregulates glycolysis, causes a loss of Foxp3 expression in Tregs and induces their conversion into effector T cells, leading to the development of autoimmune lymphoproliferative disease. Moreover, the loss of AKT activity restores the functions of *Pten*-deficient Tregs [268].

However, in contrast with these inhibitory function of PI3K, the treatment of B-cell lymphoma and leukemia patients with a highly selective PI3Kδ isoform-specific inhibitor (idelalisib, Gilead, Foster City, CA, USA) is frequently associated with immune-mediated adverse effects such as hepatotoxicity, enterocolitis, skin rash and pneumonitis associated with a reduced number of Tregs [274]. This observation thus strongly suggests a positive role of PI3Kδ in the amplification or stabilization of Tregs. This has been confirmed by the preferential inhibition of TA-Treg proliferation, signaling and suppressive function compared to effector CD4^+^ T cells after the in vitro idelalisib treatment of TILs from CLL patients [275] and in vivo in a murine model of lung cancer [276]. Mechanistically, the PI3Kδ inhibitor, by blocking the S9 phosphorylation of GSK-3b that favors GSK-3b activation, reduces the expression of MCL-1, limiting Treg cell survival. However, the role of PI3Kδ signaling differs throughout the Treg lifespan. Indeed, a reduced PI3K*δ* activity is required during development, whereas the PI3Kδ pathway is an important actor of mature Tregs immune suppression [277]. This different dependency on PI3Kδ represents an interesting therapeutic potential to selectively target highly activated effector TA-Tregs in the TME without affecting resting Tregs from the periphery.

### 4.6. CCL1/CCR8

CCR8 expression participates in Treg stability. Indeed, the engagement of CCR8 by CCL1 increases the expression of the *FOXP3* gene through the STAT3 pathway, favoring human Treg stabilization [94]. Moreover, in human MIBC tumors, CCR8 expression by TA-Tregs maintains their stability and potentiates their suppressive function by upregulating the expression of transcription factors FOXO1 and c-MAF [82], and the ex vivo blockade of CCR8 destabilizes human TA-Tregs into a fragile phenotype accompanied with the reactivation of antitumor immunity and augmentation of anti-PD-1 therapeutic benefits in MIBC [82].

### 4.7. IL-1 Receptors

The expression of IL-1 receptors by Tregs has not been widely investigated, even if scarce data has reported that, in human blood, IL-1R1 is faintly expressed on effector CD4^+^ and CD8^+^ T cells and not expressed on Tregs. Where IL-1R1 is a signaling receptor for IL-1, which mediates its function, IL-1R2 neutralizes IL-1 either as a surface decoy receptor or in a cleaved and secreted form [278,279,280]. In addition, both IL-1R1 and IL-1R2 are induced on human Tregs after TCR signaling [281]. Moreover, in the context of rheumatoid arthritis, IL-1R1 is expressed on tissue-activated Tregs [282]. Recently, in mice, Th17 polarizing conditions have been shown to favor IL-1R1 expression by Tregs. In vitro, IL-1β combined with IL-6 increases the proliferative capacity of Tregs and their survival [283] but decreases their suppressive capacity and induces the acquisition of RORγt transcription factor expression, leading to Treg differentiation into Th17 cells [283]. Consistently, in mice, IL-1R1 is expressed by unstable Tregs with reprogramming potential into Th17 cells [283].

IL-1R2, apart from its decoy receptor, function either as membrane-bound or released forms and are is also present in the cytoplasm, where they can bind pro-IL-1α, preventing its cleavage and activation [284]. It has been suggested to be linked to sensing inflammatory signals, proliferation and expansion in order to control inflammation. In mice, Helios^+^Nrp1^+^ nTregs that recirculate in the body express IL-1R2, and these IL-1R2^+^ Tregs are also detected in nonlymphoid tissue [285]. The authors also reported that IL-1β blocks intra-thymic Treg development, and that addition of IL-1R2^+^ Tregs in this inflammatory environment reverses this blockade.

Niedzielska et al. [48] recently reported the presence of *IL1R2* in the gene signature associated with resident Tregs in normal tissues, such as the lungs and colon in humans. The analysis of RNA-seq or scRNA-seq datasets from studies comparing T-cell-infiltrating tumor tissue with those from NAT and blood in breast carcinoma, CRC and NSCLC [43,44,46,79] highlighted an increased *IL1R2* gene expression on TA-Tregs. In the thymus of mice, a more in-depth phenotypic analysis of IL-1R2^+^ Tregs highlights their expression of CXCR5 and PD-1, suggesting they can be related to follicular Tregs (Tfr) that may participate in the thymus in the shaping of Treg repertoire by interacting with and modulating the function of intra-thymic B cells [285]. As tertiary lymphoid structures comprising B cells, T cells and mature DC (DC-LAMP^+^), they are present in human solid tumors and associated with a good prognosis and response to immunotherapy [286,287,288]; a better phenotypic characterization and spatial localization of the IL-1R2^+^ Tregs in the TME will help to decipher their relation to Tfr. Moreover, the characterization of IL-1R2 expression in all breast cancer subtypes by immunohistochemistry on a tumor tissue array highlighted the elevated IL-1R2 levels in tumors compared to normal tissue [289]. Moreover, the analysis of the TCGA cohort reported that breast cancer patients harboring a high *IL1R2* mRNA expression in tumors have a poorer overall survival and relapse-free survival (RFS) [289]. It could, in part, rely on IL-1R2 expression by Tregs in the TME that directly neutralizes the IL-1 (α or β) with antitumorigenic properties or stabilizes Tregs by antagonizing IL-1β-induced conversion into Th17 cells [290] or limits their own proliferation induced by IL-1β.

### 4.8. ST2/IL-33 Pathway

ST2, another member of the IL-1R family, is constitutively expressed by a subset of resident Tregs present in nonlymphoid tissues, such as adipose tissues [291], lung [292], gut [293], colon [294] and muscle [295,296]. These resident ST2^+^ Tregs are characterized by a unique transcriptional and epigenetic program (*GATA3*, *BATF* and *IRF4*) that directly regulates their differentiation, as well as ST2 expression [297]. ST2^+^ Tregs can also originate from pTregs [293,298]. In humans, IL-33, the ST2 ligand, is a nuclear cytokine constitutively expressed by epithelial/stromal cells in a large number of tissues, with an enrichment in the epithelia barrier (lungs, skin, intestines and stomach) [299,300,301,302]; endothelial cells [299] and fibroblastic reticular cells from lymphoid organs [300]. It could also be expressed by fibroblasts, myofibroblasts or various stromal cells under inflammatory conditions [303,304,305]. In response to the alarmin IL-33 released by stress in damaged tissue, ST2^+^ Tregs secrete immunosuppressive cytokines to restrict the infiltration of inflammatory cells in damaged tissue and favor tissue repair (for review [306]). In most of the human scRNA-seq studies, the *IL1RL1* gene, coding for ST2, is upregulated in TA-Tregs compared with their counterparts in NAT or blood and with other T-cell subsets, suggesting that the ST2/IL-33 pathway could play a role in TA-Treg function and/or stability in the TME [43,46,78,79,80,81]. In contrast to IL-1, a recent study in CRC reported that the IL-33/ST2 axis regulates T-cell plasticity, by stabilizing the phenotype of IL-17^neg^FOXP3^+^ Tregs and potentially promoting the conversion of IL-17-producing CD4^+^ T cells into these IL-17^neg^ (RORγt^neg^) FOXP3^+^ Tregs [294]. Similar findings have been described in autoimmune contexts, where the IL-33/ST2 axis promotes Treg stability and expansion but also favors the conversion of CD4^+^FOXP3^neg^ T cells into FOXP3^+^ iTregs [293]. Moreover, mice with *St2*-deficient Tregs display an impaired growth of transplanted tumors, suggesting a role for ST2 in Tregs’ suppressive function [79,294,307]. In addition, Hatzioannou et al. recently described a cell-intrinsic role of IL-33 in Tregs’ functional stability during tumor development; indeed, *Il-33*-deficient Tregs exhibited the impaired suppressive properties associated with the development of a robust antitumor immunity, leading to tumor eradication [308]. In *Il-33*-deficient Tregs, the TME induces an epigenetic reprogramming that maintains Foxp3 but favors a phenotype close to “fragile” Tregs by upregulating mTOR activity and IFN-γ expression but downregulating Nrp1 [268,309]. Altogether, these results suggest that the IL33/ST2 pathway delineates a molecular program orchestrating Treg stability within the TME.

### 4.9. Conclusions

Taken together, these results highlight that numerous mechanisms are involved in the stability of Tregs to favor their suppressive function in the TME. As the restriction of Tregs in the TME will efficiently participate in the reactivation of an efficient antitumor immune function in “hot” tumors, targeting one or multiple mechanisms involved in Treg stability could be helpful to transform Tregs into effector CD4^+^ T cells with antitumor properties.

## 5. Therapeutic Strategies to Block Treg Recruitment and Expansion or Limit Their Stability

All the data presented in this review illustrate a distinct contribution of particular FOXP3^+^ Treg subpopulations to the suppression of tumor immunity and, consequently, to cancer progression, indicating the importance of removing/neutralizing highly suppressive terminally differentiated effector Tregs from the tumor tissue to enhance the antitumor immune responses. Several strategies have been developed to block their recruitment and/or proliferation or to kill them according to their specific phenotype (see Table 1).

### 5.1. Inhibit Tregs Recruitment

#### 5.1.1. CCR4 Inhibitors

Several small chemical CCR4 antagonists have been reported. These allosteric inhibitors could be grouped into two classes based on their proposed binding sites. Class I antagonists bind to an extracellular region of CCR4, whereas class II antagonists bind to an intracellular region. Class II inhibitors are represented by molecules developed by AstraZeneca (Cambridge, UK) (AZD-2098 and AZD-1678) [310] or GlaxoSmithKline (GSK), (Brentford, UK) (GSK-2239633). The GSK antagonist suffered from low-target engagement and low blood exposure, which prevented further development [311]. Despite the good capacity of AstraZeneca compounds to antagonize CCR4 [312], no further development has been reported to date.

In a murine melanoma tumor model, a treatment with the CCR4 antagonist in both prophylactic and therapeutic settings [313] is more efficient than cyclophosphamide to elicit Ag-specific CD8^+^ T cells and partly inhibit tumor growth [314]. In addition, the team of Zibinsky developed new class I inhibitors [315,316,317]. As a single agent, or in combination with anti-PD-1 in preclinical in vivo studies in CRC, these CCR4 antagonists inhibit the recruitment of Tregs in the TME and elicit antitumor responses. These results have led to the development of the FLX475 molecule (Flxbio Inc (South San Francisco, CA, USA)) currently being tested in phase 1 clinical trials alone or in combination with anti-PD-1 in advanced cancer (NCT03674567). Another CCR4 antagonist, RPT193, from RAPT Therapeutics (South San Francisco, CA, USA) is currently being tested in phase 1 (NCT04271514) in the treatment of atopic dermatitis and other allergic inflammatory diseases in order to reduce the Th2 cell recruitment to inflamed lesions. However, no evaluation of cancer patients is planned to date.

#### 5.1.2. CCR8 Inhibitors

As said before, the ex vivo blockade of CCR8 in MIBC tumor suspension destabilizes TA-Tregs and favors their production of IL-17A [82]. Moreover, in murine tumor models, targeting CCR8 with a CCR8-neutralizing mAb (mIgG2b) induces a protective immunity and enhances the vaccine-induced response to CRC in CT26 and MC38 subcutaneous tumor models through the inhibition of CCR8^+^ Treg recruitment without impacting the peripheral FOXP3^+^ Treg subsets [96]. The CCR8 inhibitor (ML604086) developed by MedImmune (Gaithersburg, MD, USA) was previously developed with the aim to treat asthma marked by infiltration of a high number of eosinophils and Th2 cells responsible for the disease [318]. However, even if this compound can efficiently block the CCL1/CCR8 axis in vitro, it failed to demonstrate an efficacy in vivo in a primate model [319], thus stopping the evaluation of CCR8 inhibition for the treatment of asthma. In the same objective, an allosteric CCR8 inhibitor (AZ084) with potent selectivity profile in vitro and completely blocking DC, T cells and eosinophils migration, has been developed by AstraZeneca [320]. Even if no further data are available on this compound, the efficacy of such compounds needs to be reevaluated in the context of tumors. Interestingly, R243, a small-molecule antagonist of CCR8, enhanced the antitumoral effect of temozolomide (an antineoplastic) in a mouse model of glioblastoma [321].

#### 5.1.3. Other Chemokine Receptor Antagonists

As said before, CCR2 and CCR5 are expressed on some TA-Tregs. Thus, BMS developed a small inhibitor targeting both CCR2/CCR5 (BMS-813160), which is currently being tested in phase 1/2 clinical trials in metastatic CRC and advanced PDAC, (NCT03184870) with the objective to reduce Tregs but, also, MDSC and M2-polarized TAM recruitment and enhance antitumor immunity.

ChemoCentryx (Mountain View, CA, USA) has also developed a small-molecule targeting CCR2 (CCX-872) evaluated in phase 1 in patients with unresectable PDAC (NCT02345408), with the objective to reduce the suppressive myeloid cells in the tumor and slow the progression of disease in these patients rather than altering the Treg recruitment in the TME [356]. Noteworthy, the redundancy between the biological activity of chemokines represents an important challenge to efficiently inhibit Treg recruitment in the TME.

#### 5.1.4. TLR9 Ligand and Type-I Interferon

In murine tumor models (CRC (CT26), melanoma (B16) and PDAC (Panc02)), Anz et al. recently demonstrated that CpG treatment (TLR9 ligand) specifically reduced the tumor infiltration by Tregs without affecting spleen Tregs or other TA-T cells, indicating a specific inhibition of Treg migration into the TME [357]. This relied on a strong and specific decrease of CCL22 production by DC and TAM in the TME upon type-I IFN induced by TLR9 ligand treatment that limits CCR4^+^ Treg recruitment [357]. However, in human breast, ovarian and HNSCC cancers, our group and others have demonstrated the functional alteration of TA-pDC in their capacity to secrete IFN-α in response to TLR9 ligand, resulting from TGF-β1 and TNF-α produced in the TME [358,359,360,361]. This observation was also confirmed in mammary, cervical and melanoma murine tumor models [362,363]. This suggests in these tumors that TLR9 ligand treatment will have to be combined with inhibitors of the TGF-β pathway to restore the functionality of TA-pDC and favor type-I IFN secretion (Figure 4).

### 5.2. Inhibit Tregs Amplification in the TME

#### 5.2.1. Anti-ICOS Antagonists

Anti-ICOS antagonists have been developed in the objective to prevent interactions between ICOS^+^ Tregs and ICOS-L^+^ pDCs (Figure 4). Their use resulted in the inhibition of Treg proliferation and the blockade of IL-10 secretion by conventional CD4^+^ T cells [152,153].

MEDI-570 developed by MedImmune is a human hIgG1 directed against the ligand-binding domain of ICOS. MEDI-570 was described to induce the depletion of ICOS^+^ T cells by ADCC in monkeys [364]. This mAb is currently being tested in a phase 1 trial (NCT02520791) but restricted to patients with a relapsed/refractory peripheral T-cell lymphoma follicular variant and angioimmunoblastic T-cell lymphoma expressing high ICOS levels with the objective to directly kill tumor cells rather than depleting Tregs and reactivating effector CD4^+^ T cells.

However, Mo et al. [365] recently demonstrated, in a mouse prostate tumor model, that ICOS^+^ Treg depletion with an antagonist mAb strongly increased the efficacy of GM-CSF-modified cancer cell vaccine. In the same way, Burlion et al. recently reported, in humanized mice with breast tumors, that a combination of neutralizing anti-ICOS mAb and chemotherapy controls tumor growth by reducing the Treg proportion and increasing the CD8^+^ T cell/Treg ratio in the TME [164]. Therefore, depleting the TA-Tregs with the ICOS antagonist might be a promising immunotherapy strategy. However, as ICOS could be overexpressed on activated effector T cells after Ag recognition [366] or anti-CTLA-4 immunotherapy [367,368,369], the windows of treatment need to be restricted to the period of time when only Tregs express high ICOS levels. The development of bispecific mAbs with ICOS and other molecules specifically expressed on TA-Tregs may help to specifically target TA-Tregs without affecting highly activated effector T cells.

#### 5.2.2. Fully Human Anti-TNFR2 Antibodies

TNFR2-expressing TA-Tregs, present in the TME, participate in tumor immune evasion as TNF-α through TNFR2 and trigger their activation and proliferation. Therefore, antagonists of TNFR2 may act as ICP inhibitors to suppress TA-Tregs. Torrey et al. recently developed anti-human TNFR2 mAbs that bind and lock hTNFR2 in an inactive conformation [188]. They demonstrated that mAbs inhibited the proliferation of Tregs while promoting the proliferation of effector T cells isolated from metastatic sites (ascites) in ovarian cancer patients [188]. The National Cancer Institute (Bethesda, MA, USA) also developed a fully human TNFR2-specific mAb, produced in a defucosylated form (E4F6), capable of inducing the ADCC-dependent killing of Tregs (patent WO/2018/213064). However, TNFR2 expression on effector T cells can co-stimulate their own activation and empower their ability to resist Treg-mediated suppression [370]. Thus, the potential inhibitory effect of TNFR2 antagonists on the activation and expansion of Ag-specific effector T cells in human patients should be considered and carefully evaluated. Nevertheless, TA-Tregs are known to persistently express much higher levels of TNFR2 than their effector counterparts [183,370]. Therefore, it can be expected that the in vivo treatment with TNFR2 antagonists will have a more profound impact on TA-Tregs than on effector T cells. In sharp contrast, scientists from Merrimack Pharmaceuticals (Cambridge, MA, USA) claim that the agonism of TNFR2 at the surface of CD8^+^ T cells is the dominant mechanism of action responsible for the antitumor activity observed with their anti-TNFR2 mAbs in mouse models [371]. The results from the first clinical trial recently launched to evaluate an anti-TNFR2 antagonist (BI-1808) from BioInvent International AB (Lund, Sweden) in cancer patients (NCT04752826) should help to clarify the best way to target TNFR2 in humans.

#### 5.2.3. Restore Type-I Interferon to Inhibit Treg Amplification in TME

In murine and human breast tumors, TA-pDC present an altered capacity to produce type-I IFN in response to the TLR9 ligand [226,361,362]. Since type-I IFN inhibits Treg proliferation [372], this altered function leads to an increased Treg amplification by pDC through interaction with ICOS/ICOS-L [361]. In addition, the in vivo intra-tumoral administration of the TLR7 ligand in murine mammary carcinoma leads to TA-pDC reactivation and induces a potent curative effect [362]. These observations rationalize the use of TLR7 (MEDI-9197, MedImmune/3M) or TLR9 ligands to restore TA-pDC activation in the breast cancer environment and their type-I IFN production preventing TA-Treg expansion (Figure 4).

### 5.3. Destabilize TA-Tregs

As said before, the numerous mechanisms favoring Treg stability are activated in the TME to favor their suppressive function. The objective to destabilize TA-Tregs will efficiently participate in the reactivation of antitumor effector T cells, leading to an efficient antitumor immune function in “hot” tumors. However, almost all the molecules involved in these stabilization mechanisms are also implicated in a lot of immune and nonimmune processes, which makes their inhibition risky or are difficult to target due to their nuclear expression (Helios). Interestingly, the neutralization of IL-1R2 or CCR8 that appears more specific to TA-Tregs may be a good target to reduce their stability by favoring, for IL-1R2, their transformation into Th17 cells. However, as IL-1R2 is also expressed by other either immune (Kupffer cells, macrophages and neutrophils) or nonimmune cells (keratinocytes, pancreatic endocrine cells and mucus-secreting cells) in the body, the development of a depleting mAb is prohibited to avoid toxicity. In addition, the direct targeting of FOXP3 with an antisense oligonucleotide (AZD8701) to reduce their suppressive function is currently being evaluated in a phase 1 first-in-human clinical trial in combination with a PD-L1 blockade in solid tumors that have demonstrated a response to a prior PD-L1 treatment (NCT04504669). In preclinical studies, such molecules showed the ability to reduce the suppressive function of Tregs in in vitro suppression assays and to promote tumor regression in a syngeneic mouse model [349].

### 5.4. Favor Tregs Depletion

Among the numerous strategies developed to kill TA-Tregs, some mAbs targeting molecules more or less specifically expressed by TA-Tregs have been developed in order to specifically destroy TA-Tregs in the TME.

#### 5.4.1. CTLA-4 Blockade

The development of drugs that block CTLA-4 (ipilimumab (BMS) and tremelimumab (MedImmune)) has initiated a real revolution in the field of cancer immunotherapy. Anti-CTLA-4 mAbs were initially developed to neutralize the interaction of CTLA-4 with its ligands CD80 and CD86 to favor the interaction of these latter with CD28 to reactivate effector CD4^+^ and CD8^+^ T cells by removing the break. However, there is currently a consensus in mice for the ability of anti-CTLA-4 mAbs to deplete TA-Tregs that constitutively express high levels of membrane CTLA-4, while very little data are available in situ in human tumors. Using a mouse model with fully humanized Fcγ receptors, Arce-Vargas et al. [373] provided evidence that anti-CTLA-4 mAbs of hIgG2 and hIgG1 isotypes deplete TA-Tregs in the TME. The abrogation of Treg depletion with the hIgG2 isotype in *hFcγRIIa*-deficient mice highlighted the importance of FcγRIIa. These results confirm the concept that the strong membrane expression of CTLA-4 by TA-Tregs, specific for the TME, promotes their elimination by ADCC during treatment with anti-CTLA-4 mAbs. This depletion is associated with the proliferation of TA-effector CD4^+^ and CD8^+^ T cells and their increased production of IFN-γ, thus confirming the lifting of Treg-dependent immunosuppression. However, the humanization of FcγR in this study has been performed using endogenous promoters, and their expression pattern in murine immune cells partly differ from human ones. In this context, recent data from Wei et al. reported a difference in Treg alterations induced by anti-CTLA-4 mAbs, according to the model evaluated [369]. Anti-CTLA-4 mAb strongly reduces the proportion of Tregs in the TME from the murine melanoma tumor model (B16), whereas, in human tumors, the TA-Tregs proportion does not seem significantly impacted. However, in this study, the authors evaluated together patients treated with either ipilimumab alone or combined with anti-PD-1. Similar results were reported by Sharma’s group [374], who recently analyzed by immunohistochemistry the TME pre- and post-treatment with ipilimumab (IgG1) or tremelimumab (IgG2) in biopsies from different solid tumors (prostate and bladder carcinoma and metastatic melanoma). They reported that, contrary to mice studies, anti-CTLA-4 mAbs in humans, independently of the isotype, increases the infiltration of CD8^+^ and CD4^+^ T cells without depleting TA-Tregs. In contrast, a previous study in prostate cancers [368] demonstrated that the clinical response to ipilimumab is associated with a reduced number of TA-Tregs. In addition, a more recent study demonstrated a strong reduction in the number of TA-Tregs post-treatment in responder patients in metastatic melanoma treated with ipilimumab [375]. These disparities could result from the timing of the evaluation, as in Sharmas’ paper, where the analysis was performed on biopsies collected late post-treatment (>five weeks). In fact, the kinetic data in mice demonstrate that the depletion of Tregs is observed rapidly (two–five days) after treatment with anti-CTLA-4 mAb [373]. This depletion is probably no longer detectable five–eight weeks post-treatment because of the recruitment of unaltered Tregs. In addition, given the low percentage of responder patients, it would have been interesting, in Sharmas’ study to segregate the response to treatment, since this reduction should only be observed in responders.

Of interest, Alphamab Biopharmaceuticals (Suzhou, China) recently developed a novel bispecific domain antibody (KN046) based on human IgG1 Fc fused with anti-PD-L1 and anti-CTLA-4 Fab domains that will block both PD-L1 interaction with PD-1 and CTLA-4 interaction with CD80/CD86. The wild-type IgG1 Fc portion of KN046 preserves intact effector functions, such as ADCC, that may lead to the depletion of effector TA-Tregs [354]. This drug is currently tested in different solid tumors (TNBC (NCT03872791), esophageal SCC (NCT03927495) and NSCLC (NCT03838848)).

#### 5.4.2. Humanized Anti-CCR4 Antibody

KW-0761 (mogamulizumab), developed by Kyowa Hakko Kirin (Tokyo, Japan) is an anti-CCR4 hIgG1 that favors ADCC and is clinically approved for the treatment of relapsed refractory CCR4^+^ adult T cell leukemia/lymphoma (ATCLL) and CTCL. This mAb has been evaluated in phase 1 clinical trials in NSCLC and esophageal cancer patients and demonstrated an efficient depletion of blood Tregs but, also, of Th2 cells associated to an increase in the number of MDSC [376]. Its combination with anti-PD-1 (nivolumab, BMS) in immunotherapy-naive patients with advanced/metastatic solid tumors (PDAC and HCC) was associated with 4/15 responses in HCC and 1/15 response in PDAC patients. During treatment, effector Tregs decreased and CD8^+^ T cells in TILs increased, suggesting that a combination of anti-PD-1 mAb with mogamulizumab provides a good antitumor activity with an acceptable safety profile, which could be a potential therapeutic option in cancer immunotherapy [329]. However, another clinical trial (NCT02301130) in advanced solid tumors evaluating the combination of mogalizumab with anti-PD-L1 (durvalumab, AstraZeneca) or anti-CTLA-4 (tremelimumab, MedImmune) demonstrated a depletion of Tregs in the periphery and a reduction of TA-Tregs but did not establish a clear correlation between the clinical response and reduction of effector Tregs or baseline degree of CCR4 expression [328].

Wang et al. developed a diphtheria toxin (DT)-based recombinant anti-human CCR4 immunotoxin using a unique DT-resistant yeast *Pichia Pastoris* expression system [330]. The evaluation of this immunotoxin in monkeys demonstrated the effective depletion of CCR4^+^FOXP3^+^ Tregs, both in blood and in lymph nodes [331,332], via a mechanism that does not rely on accessory cells from the innate immune system to initiate ADCC, complement-dependent cytotoxicity, or antibody-dependent cellular phagocytosis. This depletion efficacy only lasted for approximately one week in the blood. The apparition, after approximately two weeks of auto-Ab against the CCR4 immunotoxin, strongly reduced the efficacy of the subsequent treatments. However, such a treatment could be useful in inducing an only transient depletion of effector CCR4^+^ Tregs, avoiding the risk of autoimmune diseases.

#### 5.4.3. Humanized Anti-CCR8 Antibodies

JTX-1811, recently developed by Jounce Therapeutics (Cambridge, MA, USA) in collaboration with Gilead (Foster City, CA, USA), is a humanized mAb with enhanced ADCC activity to selectively deplete immunosuppressive CCR8^+^ TA-Tregs. Experiments with a surrogate mAb specific for murine CCR8 showed good activity as a single agent or in combination with PD-1 inhibitors in anti-PD-1-resistant murine tumor models [333], suggesting that JTX-1811 would be efficient to deplete CCR8^+^ Tregs in human solid tumors in favor of the antitumor immune response. Its evaluation in a clinical trial is planned. A similar molecule currently developed by BMS (BMS-986340) is able to deplete CCR8^+^ Tregs in human tumor explants [377].

In 2020, three new human anti-hCCR8 mAbs of the IgG1 isotype engineered to enhance ADCC were reported at the SITC 2020 conference: SRF114 (Surface Oncology, Cambridge, MA, USA) [334], HBM1022 (Harbour BioMed, Hong Kong, China) [335] and FPA157 (Five Prime Therapeutics, South San Francisco, CA, USA) [336], with the in vitro preclinical results demonstrating the capacity of these mAbs to block the migration of CCR8^+^ Tregs toward CCL1 and to kill them by ADCC. This strongly demonstrates the importance of CCR8 as a therapeutic target for pharmaceutical companies, but none of them are currently being tested in phase 1 clinical trials.

#### 5.4.4. Antibodies Targeting ICOS

KY1044, developed by KyMab (Cambridge, UK), is a human IgG1κ mAb directed against the ligand-binding domain of ICOS, which can promote ADCC activity against ICOS^high^ T cells. In vitro, KY1044 depletes Tregs in ADCC assays. In contrast, when coated on plates, KY1044 induces IFN-γ production by anti-CD3/anti-CD28-stimulated effectors ICOS^+^ T cells. Moreover, in murine tumor models, KY1044 depletes TA-Tregs, improves the effector to the Tregs ratio and, also, induces the upregulation of inflammatory cytokines in vivo [378]. The differential ICOS expression levels on both subsets can explain these results: ICOS^high^ Tregs are depleted through ADCC, leading to the activation of ICOS^low^ effector CD4^+^ T cells that could be activated through an anti-ICOS agonist signal. However, in monotherapy, this mAb has shown limited antitumor activity via the abrogation of Tregs-mediated immune suppression. In this context, the clinical response to anti-CTLA-4 treatment with ipilimumab has been associated with the induction of a T-cell population that expresses ICOS [367,368,369,379,380]. Furthermore, in *Icos*-deficient mice, antitumor T-cell responses elicited by anti–CTLA-4 are significantly diminished, thereby impairing B16 melanoma rejection [381]. This suggests a potential interest to combine both treatments to reactivate the antitumor immunity.

KY1044 is currently being evaluated in phase 1/2 trials in selected advanced malignancies (NCT03829501) in combination with anti-PD-L1 (atezolizumab, Roche, Basel, Switzerland) in order to prove the efficacy of a combined Treg reduction and cytotoxic CD8^+^ T-cell reactivation in cancer immunotherapy.

#### 5.4.5. TCR-Mimic Antibody Recognizing a FOXP3-Derived Epitope

Recently, an original molecule developed by showed the potential to selectively deplete Tregs by directly targeting FOXP3. This molecule is a TCR mimic mAb; its activity relies on the TCR-like recognition of a peptide/MHC complex, allowing mAb access to intracellular Ag [382]. The mAb recognizes a human FOXP3-derived epitope in the context of HLAA*02:01 and mediates ADCC against FOXP3-expressing Tregs.

### 5.5. Risk to Reactivate TA-Tregs through Anti-ICP mAbs

#### 5.5.1. Antibodies Neutralizing PD-1/PD-L1 Interaction

The clinical authorization of biological drugs that neutralize the inhibitory PD-1/PD-L1 interaction with mAbs targeting PD-1 (nivolumab (BMS), pembrolizumab (Merck-Biopharma, Darmstadt, Germany), cemiplimab (Sanofi Aventis, Paris, France), toripalumab (Shanghai Junshi Biosciences, Shanghai, China), sintilimab (Lilly & Innovent, Indianapolis, IN, USA), camrelizumab (Jiangsu Hengrui Medicine Co, Jiangsu, China)), PD-L1 (atezolizumab (Roche), avelumab (Merck) and durvalumab (AstraZeneca)) to reactivate effector T cells blunted by the PD-L1/PD-1 interaction has initiated a real revolution in the field of cancer immunotherapy. So far, authorizations have been granted for a small number of cancers histotypes (melanoma, NSCLC, urothelial cancers and HL), but more than 2000 active clinical trials with these drugs are underway. However, as effector TA-Tregs present in the TME also express high levels of PD-1, as shown by the RNA-seq and phenotypic analyses of TA-T cells [43,46], their functions could also be modulated by anti-PD-1. Contrary to ipilimumab (IgG1), approved anti-PD-1 mAbs are all of the hIgG4 isotype to favor the neutralization of PD-L1/PD-1 interactions without depleting PD-1^+^ immune cells by ADCC. In the murine model, the engagement of PD-1 by PD-L1 was shown to stabilize Foxp3 in iTregs [383]. This effect is mediated by the activation of PTEN, which negatively regulates AKT, leading to Treg stability [384]. In contrast, in humans, Elkord’s group reported that anti-PD-1 mAb interferes with the differentiation of iTregs from naive CD4^+^ T cells, generating FOXP3^+^ Tregs with reduced suppressive function and IL-10 secretion capacity [385], whereas it does not affect the phenotype and function of nTregs [386,387]. They also suggest that the immunostimulatory effects of anti-PD-1 are mediated via the release of effector T cells from suppression. However, Sakaguchis’ group recently reported in humans and mice that PD-1 deficiency or a PD-1 blockade, in addition to increased effector T-cell activation, also increases the proliferation and suppresses the function of human nTregs [221]. This is in line with the results from Saleh et al., who demonstrated that the treatment of human primary breast tumor explants with anti-PD-1 upregulates the *CCR8* gene specifically on TA-Tregs, also suggesting an activation of TA-Tregs under treatment [388]. In addition, Sakaguchis’ group reported, in advanced gastric cancer patients who do not respond to anti-PD-1 mAb, that the frequency of proliferating (Ki67^+^) effector TA-Tregs is increased in the TME compared to patients responding to treatment. Moreover, in vitro, PD-1 neutralization increases the suppressive function of these TA-Tregs. Taken together, these results strongly suggest that anti-PD-1 treatment could, in some patients, increase the TA-Treg functionality in addition to reactivate effector T cells, then reduce the clinical benefits of the treatment. Sakaguchis’ group suggests this mechanism could participate in the development of “hyper-progressive disease” with poor clinical outcome observed in 10% of gastric cancer patients treated by anti-PD-1 [221]. In contrast, in human melanoma and murine tumor models, mass cytometry analyses used to comprehensively profile the effects of anti-PD-1 mAbs on TILs reveal an increased CD8^+^/Treg cell ratio due to an expansion of CD8^+^PD-1^+^ T cells, whereas the Tregs population was not significantly modulated [369]. These discrepancies suggest that anti-PD-1 could differently affect the TILs according to the tumor analyzed, or its efficacy could depend on the initial Tregs/effector T cells ratio in the TME.

A fusion protein composed of the extracellular domain of hTGF-β-RII (TGF-β-Trap) linked to the C-terminus of the human anti-PD-L1 heavy chain (anti-PD-L1) of the IgG1 isotype (M7824) could help to avoid such adverse effects of the PD-1/PD-L1 blockade. Indeed, PD-1^+^ effector Tregs producing TGF-β either as a secreted factor or a membrane protein [389] may be selectively inhibited either by the neutralization of their suppressive function or depletion by ADCC. Preliminary, the in vitro data in humans support this hypothesis, as an addition of M7824 in a coculture of effector CD4^+^ T cells and Tregs favors the expansion of effectors [390]. This molecule is currently being evaluated in a phase 1 clinical trial by Merck (bintrafusp-alfa) in patients with biliary tract cancer (NCT04066491 and NCT0383661) [350] and advanced NSCLC (NCT02517398) [351]. The preliminary results suggest a better overall response in patients with high PD-L1 expression on tumor cells.

#### 5.5.2. Other Antibodies Targeting Activating Receptors

- OX40 and 4-1BB agonists antibodies

The stimulation of OX40 and 4-1BB with agonists mAbs will activate effector T cells, while dampening the activity of Tregs (for review, [391]). The murine format of the first anti-hOX40 agonist (clone 9B12, mIgG1), developed by Providence Health & Services (Renton, WA, USA), was evaluated in a phase 1 clinical trial (NCT01644968). This treatment induced an increased immune activity in patient tumor samples but failed to achieve any partial responses in the advanced cancer patients participating in the study [346]. Most major pharmaceutical companies have developed at least one anti-OX40 agonist mAb (MEDI-6469 and MEDI-0562 (AstraZeneca), PF-04518600 (Pfizer, New York, NY, USA), GSK3174998 (GSK), BMS-986178 (BMS) and MOXR0916 (Roche)) currently in clinical trials alone or in combination with anti-PD-1 or anti-PDL-1 mAbs. Moreover, the Alligator Bioscience company (Lund, Sweden) recently developed a CTLA-4 x OX40 bispecific mAb based on IgG1-Fc (ATOR-1015) that induces in vitro both T-cell activation and Treg depletion. The treatment of hOX40-transgenic mice with established syngeneic tumors (bladder, CRC and PDAC) with ATOR-1015 induced tumor-specific and long-term immunological memory. Mechanistically, ATOR-1015 localized to the tumor area enriched in CTLA-4^+^ TA-Tregs and reduced their frequency but increased the number and activation of CD8^+^ T cells [355]. These results led to the development of a first-in-human clinical trial to evaluate the safety ATOR-1015 in advanced solid malignancies (NCT03782467).

The first clinical trial testing the 4-1BB agonist urelumab (hIgG4), developed by BMS in monotherapy, was put on hold until 2012 due to hepatic toxicity [392], which was recently reported as resulting from the S100A4 protein secreted by tumor and stromal cells [393]. More recent studies have reported the safety of this mAb, even if no specific trial has been developed as a monotherapy recently [348]. The results from a phase 1 clinical trial (NCT01307267) evaluating another anti-4-1BB (utomilumab, hIgG4) developed by Pfizer reported a favorable safety profile and preliminary antitumor activity, warranting further evaluation in patients with advanced malignancies. However, none of these trials analyzed the modulation of Tregs under treatment.

However, apart their agonist impacts on effector CD4^+^ and CD8^+^ T cells favoring an antitumor immune response, these anti-4-1BB and OX40 agonists mAbs could also favor the amplification of TA-Tregs expressing high levels of these molecules.

- ICOS agonist antibodies

The costimulatory signal of ICOS/ICOS-L is widely involved in antitumor T-cell responses. Nevertheless, ICOS signaling might have pro-tumoral features, due to its high expression on TA-Tregs. While the presence of ICOS^+^ TA-Tregs in TILs were correlated with a poor prognosis in human tumors, effector CD4^+^ICOS^+^ T cells were found to be enriched in the peripheral blood and tumor tissues of patients treated with anti-CTLA-4 antagonistic mAbs. Therefore, agonist anti-ICOS mAbs was developed by GSK (GSK3359609) and Jounce Therapeutics (JTX-2011) and evaluated in phase 1 open-label studies alone and in combination with anti-PD-1 mAbs in subjects with selected advanced solid tumors (INDUCE-1, NCT02723955 and ICONIC, NCT02904226) [341,343] to stimulate the amplification of these effector CD4^+^ ICOS^+^ T cells. The latest data reported reveal a good tolerance profile in monotherapy or in combination with nivolumab. GSK3359609 is currently being evaluated in phase 2/3, in combination with pembrolizumab, in HNSCC patients with PD-L1-positive scores (INDUCE-3, NCT04128696), whereas JTX-2011 (vopratelimab) is currently being evaluated in a phase 2 study in combination with the anti–PD-1 monoclonal antibody JTX-4014 in patients with NSCLC who have not received immunotherapy (SELECT, NCT04549025). However, these agonist mAbs might be highly deleterious in an environment enriched in ICOS^+^ TA-Tregs, as it might also stimulate their amplification [394]. Thus, it would be very important to investigate the ratio of ICOS^+^ TA-Tregs to effector ICOS^+^CD4^+^ T cells in the TME before initiating such studies.

It would also be important to scrutinize the immuno-monitoring results of the first clinical trials evaluating the numerous anti-TIM-3 (TSR-022 (Tesaro,,Boulogne Billancourt, France), MBG453 (Novartis, Basel, Switzerland), LY3321367 (Lilly), Sym023 (Symphogen/Servier, Ballerup, Denmark), anti-TIGIT (vibostolimab (Merck) and tiragolumab (Genentech, South San Francisco, CA, USA)) antagonist mAbs to confirm they will not favor Treg expansion.

## 6. Conclusions and Perspectives

The mechanisms of Treg recruitment in the TME are quite well-established, leading to identifying the targets well, such as CCR4 and CCR8, pursued for drug development. However, the use of mAbs blocking their recruitment versus those favoring their specific depletion in the TME (in particular, for those targeting CCR8 mAbs) remains a dilemma. The current strategy developed by all the current pharma deal with the use of depleting mAbs (Table 1). However, it may lead to the rapid apoptosis of TA-Tregs at the tumor site, which will therefore modify the TME by favoring the recruitment of other immune cells with potential pro-tumoral functions (neutrophils). Furthermore, although CCR8 is the chemokine receptor the most specific for TA-Tregs, the depletion of other CCR8^+^ cells may have higher side effects compared to CCR8 neutralization.

In addition, other aspects of TA-Treg biology in the TME need further investigations. Where their high expression of activating receptors such as ICOS and TNFR2 is well-demonstrated, leading to the development of drugs with the aim to specifically deplete/neutralize them, the expression of others such as OX40, 4-1BB and GITR requires further investigations, as these receptors, also expressed by effector T cells, are currently targeted through agonist mAbs. Concerning the inhibitory receptors, TA-Tregs express high levels of PD-1, CTLA-4 and TIGIT that could be targeted. However, in contrast to anti-CTLA-4 (hIgG1) aiming to deplete highly activated TA-Tregs in the TME, the inhibition of such inhibitory ICPs with nondepleting mAbs (anti-PD-1, hIgG4) may increase their suppressive function by releasing a brake. Moreover, the comprehension of the mechanisms involved in the stability of Tregs (PI3K, IL-1R2 and NRP1.) represents another field to explore in order to specifically reduce the TA-Treg frequency by favoring their plasticity.

Several therapeutic strategies based on the known suppressive function of Tregs (CD39/CD73/Ado, TGF-β and CTLA-4 pathways) are under clinical development, but because Tregs exert their suppressive function through multiple pathways, their clinical benefits will likely be restricted to specific contexts. In contrast, strategies allowing to selectively deplete or block Treg recruitment and their expansion at the tumor site or destabilize them will interfere with all their suppressive mechanisms, including the amplification of the inhibitory activity of myeloid cells.

The current new developments in the field of Tregs in oncology aim to design drugs more selective for TA-Tregs with the generation of bispecific mAbs such as bintrafusp-alfa (TGFβ trap/anti-PD-L1 and ATOR-1015 (anti-CTLA-4/anti-OX40) or KN046 (anti-PD-L1/anti-CTLA-4) or to develop treatments combining TA-Treg blockades or depletion (anti-CTLA-4, anti-CCR8, anti-ICOS and anti-TNFR2) and effector T-cell reactivation with agonist mAbs (anti-ICOS, anti-OX40, anti-4-1BB, etc.). Such drugs will help to reduce the severe adverse effects due to the nonselective disruption of Tregs throughout the body, favoring autoimmune manifestations.

## Figures and Tables

**Table 1:** Different therapeutic strategies developed to antagonize regulatory T-cell (Treg0 immunosuppression in tumors.

Target	Name	Producer	Description	Mode of Action	Stage of Development	Clinical Trial Register	Ref.
**CCR4**	AZD-2098	AstraZeneca	Small chemical class-II antagonist	Blocks chemokine-induced cellular response (Ca^++^ influx) Blocks Treg recruitment	Marketed		[310]
	AZD-1678	AstraZeneca	Small chemical class-II antagonist	Blocks Treg recruitment	Preclinical studies		[310]
	FLX475	FlxBio Inc.	Small chemical class-II antagonist	Prevents link between CCL22 and CCR4 (competition) Blocks Treg migration to the TME	Phase 1/2: Monotherapy or combination with anti-PD-1 (Merck Biopharma (Merck)) (advanced cancers)	NCT03674567	[322,323]
	KW-0761	Kyowa Hakko Kirin	Humanized mAb (defucosylated hIgG1)	Supports ADCC by linking to CCR4	Phase 1a: Monotherapy (solid tumors)	NCT01929486	[324,325]
					Phase 1/2: Combination with anti-PD-1 (Merck) (B-cell lymphoma)	NCT03309878	[326]
					Phase 1b: Combination with anti-4-1BB (Pfizer) (advanced solid tumors)	NCT02444793	[327]
					Phase 1b: combination with anti-PD-L1 or anti-CTLA-4 (AstraZeneca) (advanced solid tumors)	NCT02301130	[328]
					Phase 2: combination with anti-PD-1 (BMS) (advanced solid tumors)	NCT02476123	[329]
	anti-CCR4-DT immunotoxin	Harvard Medical School	Single-chain fold back diabody anti-hCCR4 coupled to DT	Binds CCR4^+^ cells Inhibits cell proliferation and protein synthesis ADCC, CDC and ADCP	In vitro studies in human PBMC Preclinical studies in monkey		[330,331,332]
**CCR8**	AZ084	AstraZeneca	CCR8 allosteric antagonist	Blocks LT, DC and eosinophils migration	Preclinical studies		[320]
	JTX-1811	Jounce Therapeutics	Humanized mAb (hIgG1)	CCR8^+^ Tregs depletion by ADCC	Preclinical studies		[333]
	SRF114	Surface Oncology	Humanized mAb (hIgG1)	CCR8^+^ Tregs depletion by ADCC Blocks CCR8^+^ Tregs migration	Preclinical studies		[334]
	HBM1022	Harbour Biomed	Humanized mAb (hIgG1)	CCR8^+^ Tregs depletion by ADCC Blocks CCR8^+^ Tregs migration	Preclinical studies		[335]
	FPA157	Five Prime Therapeutics	Humanized mAb (hIgG1)	CCR8^+^ Tregs depletion by ADCC Blocks CCR8^+^ Tregs migration	Preclinical studies		[336]
	25B3	BMS	Humanized mAb (non fucosylated hIgG1)	CCR8^+^ Tregs depletion by ADCC	Preclinical studies		[337]
**CCR2/** **CCR5**	BMS-813160	BMS	Small inhibitor	Reduced Tregs, MDSC and TAM recruitment	Phase 1b/2: Monotherapy or combination with anti-PD-1 (BMS) (CRC/PDAC)	NCT03184870	[338]
	CCX872	ChemoCentryx	Small inhibitor	Reduces the presence of suppressive myeloid cells	Phase 1b: monotherapy (PDAC)	NCT02345408	[339]
**ICOS**	KY1044	KyMab	Human mAb (hIgG1k)	Depletes Tregs by ADCC (in vitro) Enhances production of IFN-γ by CD4^+^ Teff	Phase 1/2: monotherapy or combination with anti-PD-L1 (Merck) (advanced tumors)	NCT03829501	[340]
	GSK3359609	GSK	Humanized anti-hICOS (hIgG4)	Stimulates ICOS^+^CD4^+^ Teff	Phase 1/2: monotherapy or combination with anti-PD-1 (Merck) (neoplasms)	NCT02723955	[341]
	GSK3359609	GSK	Humanized anti-hICOS (hIgG4)	Stimulates ICOS^+^CD4^+^ Teff	Phase 2/3: Combination with anti-PD-1 (Merck) (HNSCC)	NCT04128696	[342]
	JTX-2011	Jounce Therapeutics	Humanized anti-hICOS (hIgG4)	Stimulates ICOS^+^CD4^+^ Teff	Phase 1/2: Combination with anti-PD-1 or anti CTLA-4 (BMS) Phase 2: Combination with anti-PD-1 (Merck/BMS)	NCT02904226 NCT04549025	[343]
**TNFR2**	E4F6	National Cancer Institute	Fully-human defucosylated mAb (hIgG1)	Induces TNFR2^+^ Treg killing through ADCC			[344]
	BI-1808	BioInvent International AB	Fully human mAb (hIgG1)	Combines Treg depletion, CD8^+^ T cell expansion and modulation of TA-myeloid cells	Phase 1/2a: Monotherapy or combination with anti-PD-1 (Merck) (advanced tumors)	NCT04752826	[345]
**OX40**	9B12	Providence Health & Service	Murine anti-hOX40 mAb (mIgG1)	Increases immune cell activity	Phase 1: Monotherapy in advanced cancers	NCT01644968	[346]
	MEDI-16469	MedImmune	Murine anti-hOX40 mAb	Increases Ki67^+^ ICOS^+^ CD4^+^ and CD8^+^ Teff	Phase 2: Combination with stereotactic radiation and/or chemotherapy	NCT01642290 NCT01303705	[347]
**4-1BB** **/CD137**	PF-05082566	Pfizer	Humanized anti-h4-1BB (hIgG2)	Favors anti-tumor immune response though Teff	Phase 1: Monotherapy or combination with anti-hCD20 (GenenTech) (NHL)	NCT01307267	[348]
**FOXP3**	AZD8701	AstraZeneca	Antisense oligonucleotide	Deletes FOXP3 expression on Tregs	Phase 1: Monotherapy (solid tumors) or combination with anti-PD-L1 (AstraZeneca) (TNBC, RCC)	NCT04504669	[349]
**Bi-specific mAbs**	M7824	NIH	Bifunctional fusion protein (hIgG1 anti-PDL-1 fused with 2 extracellular domains of TGFβRII)	Neutralizes and depletes (ADCC) suppressive function of PD-1^+^ Tregs producing TGFβ	Phase 1: Monotherapy in biliary tract carcinoma	NCT03833661	[350]
					Phase 1: Monotherapy in advanced NSCLC	NCT02517398	[351]
					Phase 2: Combination with chemotherapy (NSCLC)	NCT03840915	[352]
					Phase 2: platinum experienced cervical cancer	NCT04246489	[353]
	KN046	Alphamab Bio Pharmaceuticals	bispecific hIgG1-Fc fused with anti-PD-L1 and anti-CTLA-4 Fab domains	Blocks both PD-L1/PD-1 and CTLA-4/CD80-CD86 interactions	Phase 1b/2: Monotherapy or combination with chemotherapy (TNBC) Phase 2: Combination with chemotherapy and palliative radiotherapy Phase 2: Advanced NSCLC	NCT03872791 NCT03927495 NCT03838848	[354]
	ATOR-1015	Alligator Biosciences	bispecific hIgG1-Fc [Ig-like-V-type domain of hCD86 + agonist OX40 mAb]	Induces T cell activation and Treg depletion	Phase 1: Monotherapy in solid tumors	NCT03782467	[355]
